# Isolation and characterization of extracellular vesicles in saliva of children with asthma

**DOI:** 10.20517/evcna.2020.09

**Published:** 2021-03-30

**Authors:** Nicole Comfort, Tessa R. Bloomquist, Alex P. Shephard, Carter R. Petty, Amparito Cunningham, Marissa Hauptman, Wanda Phipatanakul, Andrea Baccarelli

**Affiliations:** ^1^Department of Environmental Health Sciences, Columbia University Mailman School of Public Health, New York, NY 10032, USA.; ^2^NanoView Biosciences, Malvern Hills Science Park, Malvern, Worcestershire WR14 3SZ, UK.; ^3^Brigham and Women's Hospital, Boston, MA; Boston Children's Hospital, Clinical Research Center Boston, MA 02115, USA.; ^4^Harvard Medical School, Boston, MA 02115, USA.; ^5^Division of Allergy and Immunology, Boston Children’s Hospital, Boston, MA 02115, USA.

**Keywords:** Extracellular vesicles, exosome, TEM, NTA, SP-IRIS, saliva, asthma, biomarker

## Abstract

**Aim:**

To confirm the presence of extracellular vesicles (EVs) in cell-free saliva (CFS) of children with asthma and describe the isolated EV population.

**Methods:**

A pooled sample of CFS EVs isolated from 180 participants using ExoQuick-TC was examined in downstream analyses. Transmission electron microscopy (TEM) was used to confirm the presence of EVs. Nanoparticle tracking analysis (NTA) and single particle interferometric reflectance imaging sensing (SP-IRIS) with fluorescence were used for sizing, counting, and phenotyping of EVs. Capillary immunoassays were used for protein quantitation.

**Results:**

TEM confirmed the presence of EVs of diverse sizes, indicating the prep contained a heterogeneous population of EVs. Capillary immunoassays confirmed the presence of EV-associated proteins (CD9, CD63, CD81, ICAM-1, and ANXA5) and indicated limited cellular contamination. As others have also reported, there were discrepancies in the EV sizing and enumeration across platforms. Fluorescent NTA detected particles with a mode diameter of ~90 nm, whereas SP-IRIS reported sizes of ~55-60 nm that more closely approximated the TEM results. Consistent with protein immunoassay results, SP-IRIS with fluorescence showed that the majority of these EVs were CD9- and CD63-positive, with little expression of CD81.

**Conclusion:**

EVs from CFS can be isolated using a high-throughput method that can be scaled to large epidemiological studies. To our knowledge, we are the first to characterize CFS EVs from patients with asthma. The use of CFS EVs as potential novel biomarkers in asthma warrants further investigation and opens a new avenue of research for future studies.

## INTRODUCTION

Asthma is the most common noncommunicable childhood disease, affecting approximately 8% of children worldwide and 12%-15% of urban children in the United States^[1,2]^. Asthma is a multifactorial disorder characterized by inflammation, airway remodeling, and airway hyperresponsiveness. While the clinical presentations may appear similar, evidence suggests that asthma may not be a single disease but rather encompasses a group of heterogeneous endotypes with various etiologies and prognoses^[3,4]^. Identifying biomarkers that can profile clinical subtypes early in the course of asthma is critical in applying tailored therapy, yet the utility of current biomarkers is very limited because they are either invasive or non-specific and therefore unable to faithfully identify clinically meaningful subtypes^[5]^. Thus, identification of objective biomarkers of asthma subtypes is a clinical research priority that may also advance our understanding of the various underlying pathologies that give rise to asthma.

Extracellular vesicles (EVs) are attractive candidates for biomarkers of asthma endotypes. EVs and their cargo have been implicated in asthma^[6-9]^. Also, EVs have been found in nearly all biofluids tested, including saliva^[10-12]^. Saliva, regarded as the “mirror of the body”, is an easily accessible biofluid that harbors constituents that provide sources for monitoring of health and disease states^[13,14]^ including asthma^[15-21]^. Yet, to the best of our knowledge, no study has investigated the characteristics of saliva EVs among patients with asthma. Hence, the aim of the present study is to describe the EVs isolated from cell-free saliva (CFS) of children with asthma.

## METHODS

### Study population

This study includes 180 children aged 4 to 14 years with asthma who were enrolled in the School Inner-City Asthma Study (SICAS). Details of the cohort have been described previously^[22]^. Inclusion criteria included: (1) history of physician-diagnosed asthma; (2) evidence of active asthma (defined as cough, wheezing, shortness of breath, whistling in the chest in the past year or daily controller medication use); (3) at least one unscheduled medical visit for asthma in the past year; and (4) attendance in schools that agreed to participate in the study. Written informed consent was obtained from each participant’s parent or legal guardian, and assent was obtained from each participant. The protocol was approved by the Boston Children’s Hospital and Columbia University Institutional Review Boards (Columbia IRB: AAAS9936).

### Saliva collection

Whole saliva was collected from each participant. The collection required the participant to chew on parafilm (thin paraffin wax) and continue swallowing saliva normally for 1 min. Then, participants would “hold” saliva (i.e., stop swallowing) and spit oral fluid into a cold 50 mL falcon tube every 30 seconds until at least 5 mL saliva was collected or 15 minutes of chewing/spitting had passed, whichever occurred first. The whole saliva was aliquoted into microcentrifuge tubes and centrifuged for 20 min at 13,000 × g at 4ºC to obtain CFS. Resulting CFS was removed, aliquoted (0.3-1.2 mL), and immediately stored at -80ºC until EV isolation.

### EV isolation

The exosomal fraction from CFS aliquots was isolated using ExoQuick-TC (EQ) (System Biosciences, Palo Alto, CA, Catalog No.: EXOTC50A-1) following the manufacturer’s recommendations, with some modifications for saliva. CFS was mixed with EQ in a 2:1 CFS-to-EQ volume ratio, as done previously^[23]^. Tubes were inverted 5 times and then incubated upright overnight at 4ºC. The next day, the samples were centrifuged at 1500× g for 30 min, and then 16,000 × g for 2 min. The supernatant (SN) was removed from samples and pooled. Samples then underwent a second round of centrifugation at 1500× g for 5 min, SN was removed, and samples underwent a final centrifugation of 16,000× g for 1 min. Any residual SN was removed and discarded. The addition of short, high-speed centrifugation steps enabled creation of a denser (i.e. not easily disturbed) pellet. All centrifugation steps were performed at 4ºC. The resulting pellets were re-suspended in 105 μL of 3 kDa filtered 1× Dulbecco’s phosphate buffered saline (dPBS). To create a representative sample for downstream analyses, we removed 5 μL of sample from each re-suspended EV pellet and pooled to create a single total EV sample representative of the study population. The pooled EV and SN samples were both diluted at 1:1 with filtered 1× dPBS prior to downstream analyses. The EV sample was sent directly for transmission electron microscopy and then frozen at -80ºC before further analyses (nanoparticle tracking analysis, Exoview, protein capillary immunoassay). 

### Transmission electron microscopy

5 μL of the pooled EV sample was layered onto a formvar/carbon-coated grid and allowed to settle for 60 sec. The sample was blotted and negative-stained with 4 successive drops of 1.5% uranyl acetate in water, blotting between each drop. The grids were blotted and allowed to air dry at room temperature. Grids were imaged with a JEOL JSM 1400 (JEOL, USA, Ltd, Peabody, MA) transmission electron microscope operating at 100 kV. Images were captured on a Veleta 2K × 2K CCD camera (Olympus-SIS, Munich, Germany). 10-15 images were captured from each of three randomly selected areas of each grid at 50,000× and 100,000× lens magnification. The camera magnifications were calibrated using a grid with a grating replica (EMS cata # 80050) with line spacing of 463 nm (2160 lines/mm). Scale bars reflect the magnification at the camera. Transmission electron microscopy (TEM) micrographs were analyzed manually. Rounded or “cup-shaped” particles with high-contrast edges were considered EVs. EV diameters (*N* = 70 vesicles) were measured using ImageJ 1.53a with Java 1.8.0_172 (64-bit). 

### Nanoparticle tracking analysis

#### NanoSight LM10

Nanoparticle tracking analysis** (**NTA) was performed on the NanoSight LM10 (Malvern Panalytical, Malvern, UK) equipped with a 488 nm blue laser and NTA software, Version 2.3 Build 2.3.5.0033.7-Beta7. EVs were diluted at 1:1000 in PBS (viscosity 0.97 cP) and NTA was performed at 21.4ºC on the NanoSight LM10 with the capture and analysis settings described in Supplementary Table 1. Monodisperse 100 nm polystyrene latex spheres (Colloidal Metrics, Mountain View, CA, USA) were run before measurements to ensure instrument calibration [Supplementary Figure 1]. For fluorescent NTA, the sample was stained using the ExoGlow-NTA Fluorescent Labeling Kit (System Biosciences, Palo Alto, CA, USA, Catalog No.: EXONTA200A-1) following the manufacturer’s protocol before measurement on the NanoSight. In brief, EVs are added to a buffer containing a proprietary labeling dye. The sample is mixed and incubated at room temperature for 3-5 min. Free dye is removed by passing the sample through a column. Then, labeled EVs are ready for NTA analysis. Three technical replicates were run for both conventional light scatter NTA and fluorescent NTA. 

#### ViewSizer 3000

NTA was also performed using a second instrument, the ViewSizer 3000 (Horiba Ltd, Kyoto, Japan, Software version 1.9.0.3019/1.0.9 WeekBuild 2919). The optical system of the ViewSizer 3000 makes it advantageous compared to other NTA instruments; it combines three laser light sources (450 nm, 520 nm, and 635 nm) to enable detection and recording of scattered light from individual particles simultaneously in multiple spectral bands^[24]^. The total EV sample was diluted at 1:8000 with 3 kDa filtered 1X dPBS and loaded into a cuvette and into the ViewSizer 3000. Twenty-five videos (30 frames per second, 300 frames per video) were recorded at 22ºC with the following recording parameters: Blue laser, 210 mW; green laser, 12 mW; red laser, 8 mW; exposure, 15 ms; camera gain, 30 dB. Samples were processed with the Main Chart in “LogBinSilica” and integrated in the range [50, 1900] nm. 100 nm uniform polystyrene beads (3100A Nanosphere Size Standard, ThermoFisher Scientific, Waltham, MA, USA) were run before measurements to ensure the instrument was properly calibrated [Supplementary Figure 1]. Two technical replicates of 25 videos each were run. The number of completed tracks for all NTA measurements (on both the NanoSight LM10 and ViewSizer 3000) always exceeded the proposed minimum of 1000 to minimize artifactual spikes in the data based on single large particles^[25]^.

### Capillary western immunoassay

Protein concentration of the pooled EV sample and pooled SN sample was quantified with the Nanodrop (Implen NanoPhotometer P300, Thermo Fisher Scientific, Waltham, MA, USA). We used the ProteinSimple capillary immunoassay (Wes) method following the manufacturer’s instructions (ProteinSimple, San Jose, CA, USA). Samples were frozen at -80ºC until they were sent to RayBiotech (Peachtree Corners, GA) for analysis via the Auto Western testing service. The EV sample was diluted 2X so that all samples were loaded at 1 mg/mL. All target proteins and controls were detected with antibodies provided by RayBiotech (Peachtree Corners, GA, USA). The CD9 antibody used was a mouse monoclonal IgG1k antibody with immunogen mouse CD9. The CD63 antibody used was a rabbit polyclonal antibody with immunogen AA 103-203 of recombinant human CD63 (accession #: P08962). The CD81 antibody used was a rabbit polyclonal antibody with AA 113-201 of recombinant CD81 as the immunogen (accession #: NP_004347.1). Samples were loaded with no boil/no DTT. 

### Single particle interferometric reflectance imaging sensing

#### Sample preparation

Samples were analyzed using single particle interferometric reflectance imaging sensing (SP-IRIS) using the ExoView Human Tetraspanin Kit (NanoView Biosciences, USA). 1 μL of each sample was diluted in 99 μL of manufacturer supplied buffer, solution A. 35 μL of the diluted sample was incubated on one ExoView Tetraspanin Chip per sample overnight (16 h) at room temperature. Chips were washed three times in solution A prior to incubation with fluorescent tetraspanin antibodies. The same antibody clones were used for capture and fluorescent detection (CD9: HI9a; CD63: H5C6; CD81: JS-81).

Labeling antibodies consisted of anti-CD9 CF488, anti-CD81 CF555, and anti-CD63 CF647. For fluorescent staining, 250 μL of the fluorescent antibody cocktail (antibodies diluted at 1:500 in the fluorescent cocktail) was added to 250 μL of solution A which remained on the chip post-wash (resulting in a 1 in 2 dilution of the antibody cocktail on the chip, 1:1000 dilution overall). The fluorescent antibodies incubated on chips for 1 hour at room temperature. Chips were then washed in kit-supplied buffers, dried, and imaged by the ExoView R100 using nScan v2.9.3.

#### Measurement details

Interferometric measurements for particle sizing were acquired using a 405 nm LED. The excitation and emission [Em] wavelengths of the three fluorescent channels are shown in Supplementary Table 2. CF647 was conjugated to CD63 [Absorption (Abs) max: 650 nm; Em max: 665 nm]. CF555 was conjugated to CD81 (Abs max: 555 nm; Em max: 565 nm). CF488 was conjugated to CD9 (Abs max: 490 nm; Em max: 515 nm). 

#### Data processing

Background scans of the chips were performed prior to sample incubation. Detected particles were subtracted from the particle counts post sample incubation to account for debris on the chip. For fluorescent analysis, the MIgG spots on each chip were used as a negative control to account for non-specific fluorescent antibody binding. The fluorescence intensity of particles present on the MIgG spots was used to set a baseline intensity value; only particles which exceed this fluorescence intensity were counted as positive. Thus, fluorescent cut offs were set relative to the MIgG control.

A 150-μm-diameter area of each capture spot was selected for analysis using an automated circle finding algorithm. The particles within this area were counted, producing a particle value that represents normalization of particle count to spot area. Each chip has the antibody capture spots in triplicate. Data were analyzed using NanoViewer 2.9.3.

### Statistical analyses

Statistical analysis was performed with R software^[26]^, version 4.0.3. As NTA sizing and concentration values derived from one sample are normally distributed, unpaired two-tailed Student’s *t*-test was used to compare particle concentrations and size summary statistics with alpha set to 0.05.

## RESULTS

### Evidence of EVs in human saliva of children with asthma

This analysis includes 180 children with asthma aged 4 to 14 years who were enrolled in SICAS and provided a saliva sample at the study baseline visit. Details of the cohort have been described previously^[22]^. Table 1 presents characteristics of the study population included in this analysis. The mean age of the participants was 8.1 years, and the majority (58%) of participants identified as Hispanic/Latino. 20% of participants lived in a household that reported an annual household income of less than $25,000. 22% of participants reported a smoker at home, and a majority of participants (60%) were sensitized to at least one allergen [Table 1]. 

**Table 1:** Characteristics of the SICAS study population

	**All study participants, no. (%)**
Population	*N* = 180 *
Age (years), mean (range)	8.1 (4-14)
Female sex	85 (47%)
BMI (kg/m^2^), mean (range)	19.3 (13.7-37.2)
Race/ethnicity	
Black/African American	30 (17%)
Hispanic/Latino	104 (58%)
White	26 (15%)
Mixed/other	19 (11%)
Environmental tobacco smoke	39 (22%)
Use of controller medication	103 (58%)
Atopic asthma (any allergen sensitization)	107 (60%)
Annual household income	
< $25,000	36 (20%)
≥ $25,000	84 (47%)
Missing	59 (33%)

*180 SICAS participants provided saliva samples. One participant was missing all demographic data. SICAS: School Inner-City Asthma Study.

**Figure 1:**
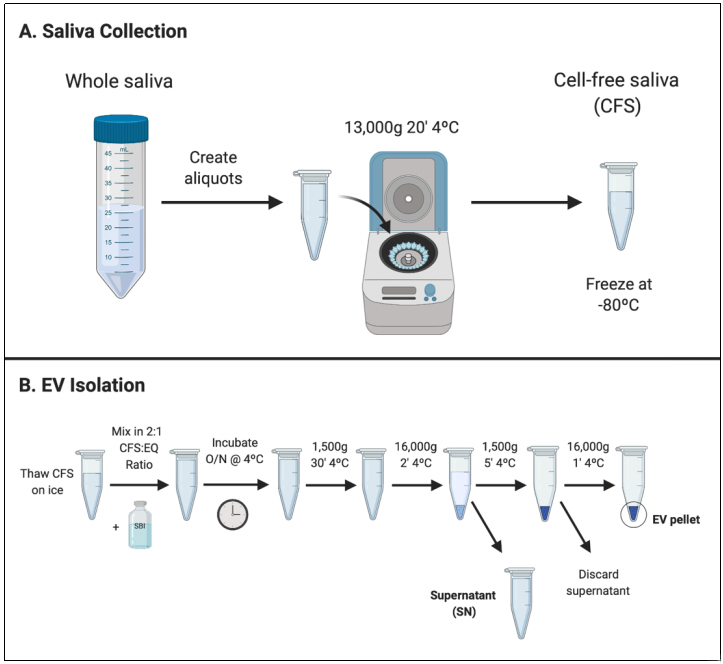
Graphical overview of laboratory methods for CFS collection and EV isolation. (A) CFS collection protocol. CFS was collected from *N* = 180 participants of the School Inner-City Asthma Intervention Study. Aliquots of CFS were frozen at -80ºC before shipment to Columbia University for EV isolation; (B) EV isolation protocol. EVs were isolated from CFS using a polymer-based reagent, EQ, in a 2:1 CFS-to-EQ ratio. After overnight incubation, EVs were precipitated from the solution by centrifugation. CFS: Cell-free saliva; EV: Extracellular vesicle; EQ: ExoQuick-TC; SBI: System Biosciences.

**Figure 2:**
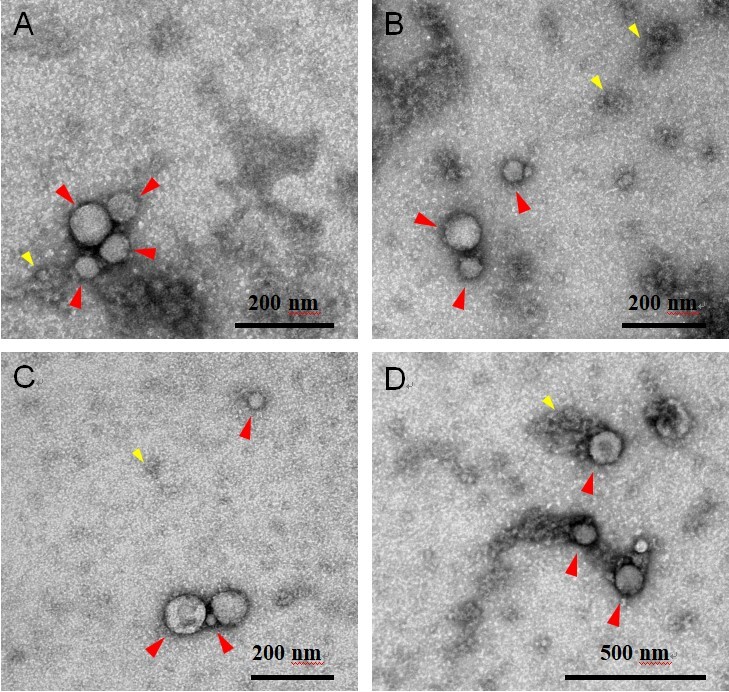
Representative electron microscopy negative staining images of pooled CFS EVs (independent EV isolations *N* = 180) showing round-shaped EVs. Electron micrographs show round-shaped vesicles (red arrowheads) 16-320 nm in diameter (mean 64.95 nm). Other structures seen in the background (yellow arrowheads) could be protein aggregates. Scale bars reflect the magnification at the camera. (A-C) 100k magnification, scale bar 200 nm; (D) 50k magnification, scale bar 500 nm. CFS: Cell-free saliva; EV: extracellular vesicle.

**Figure 3:**
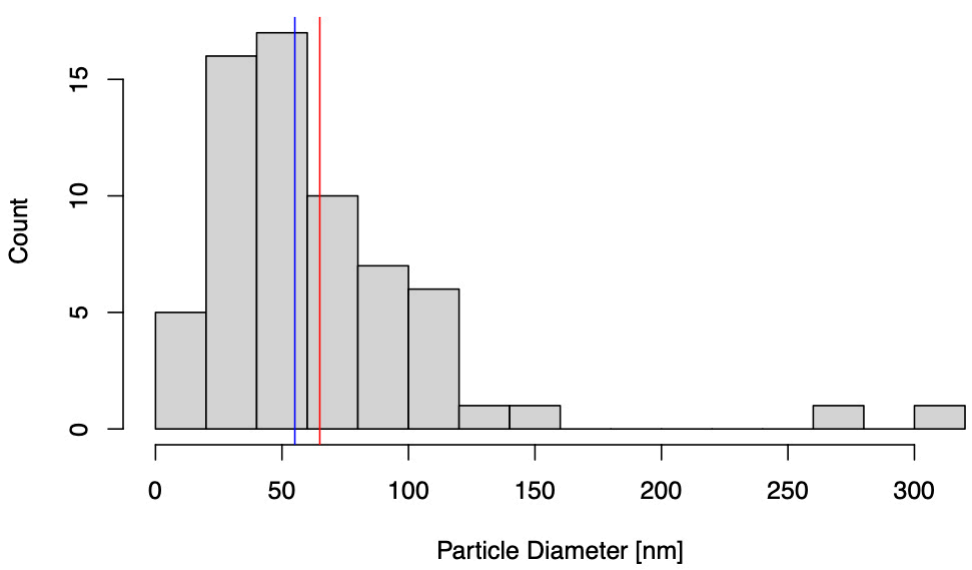
Histogram of transmission electron microscopy (TEM) particle diameters. Particle size distribution of *N* = 70 vesicles sized using ImageJ. Blue and red lines mark the median and mean particle diameter [nm], respectively.

EVs were isolated from the CFS fraction of each participant (*N* = 180) using ExoQuick-TC (EQ). An overview of the saliva collection and EV isolation protocols is depicted in Figure 1. To create a representative sample for downstream analyses, we pooled 5 μL from each re-suspended EV pellet to create a single total EV sample representative of the SICAS study population. To assess the purity of the total EV sample and characterize single vesicles, we imaged EVs using negative-stain TEM. For the TEM analysis, the pooled EV sample was examined in the Microscopy and Image Analysis Core, Weill Cornell Medicine (see Acknowledgements). Sizing analysis on vesicles (*N* = 70) was performed using ImageJ. TEM confirmed the presence of EVs of diverse particle sizes. Electron micrographs show rounded, electron-dense vesicles (Figure 2, examples marked with red arrowheads) with a mean (median) size of 64.95 (55.09) nm in diameter [Figure 3, Table 2], similar to previous reports of saliva exosomes^[10]^. Other structures present in the background (Figure 2, examples marked with yellow arrowheads) could represent protein aggregates. In rare instances, fibrous-like shapes could be seen in the micrographs, likely resulting from carry-over of the EQ solution [Supplementary Figure 2].

**Table 2:** Summary statistics of EV* measurements

	**Size (nm)**
Min	16.16
Max	320.79
Mean	64.95
Median	55.09

*EVs (*n* = 70) were sized using ImageJ software. EVs: extracellular vesicles.

### Nanoparticle tracking analysis of saliva EVs

**Figure 4:**
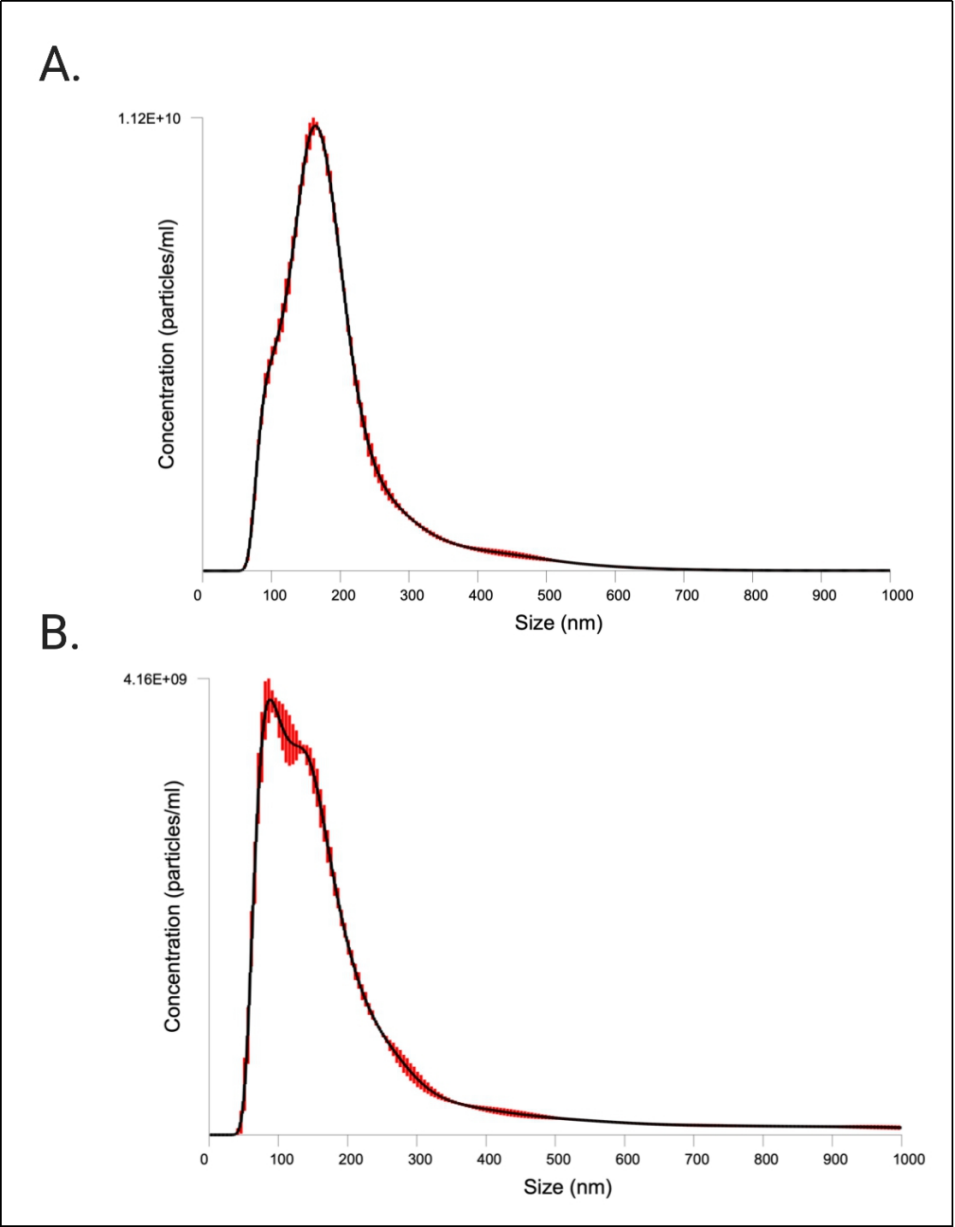
Characterization of CFS EVs by NTA. PSDs from NTA FTLA data on the NanoSight LM10 are shown. Red bars indicate ± standard error of the mean. The PSDs are the average size/concentration of three technical replicates. Graphs are representative of measuring samples using the optimal particle/frame rate according to the operating manual for NanoSight LM10. (A) NTA in light scatter mode (conventional NTA); (B) NTA in fluorescent mode (fNTA). CFS: Cell-free saliva; EVs: extracellular vesicles; NTA: nanoparticle tracking analysis; PSD: particle size distribution; FTLA: finite track length adjustment.

EV sizing and enumeration was performed using NTA on the NanoSight LM10 equipped with a blue laser (488 nm). Particle size distributions (PSDs) of the EV prep are depicted in Figure 4. According to the NTA measurements, the isolated particles were within the expected size range for extracellular vesicles, 70-404 nm. A summary of the EV concentration and size distribution, with or without the finite track length adjustment (FTLA) algorithm, is provided in Table 3. We also performed fluorescent NTA (fNTA), since conventional NTA does not distinguish membranous vesicles from co-isolated non-membranous particles of similar sizes. We found that fNTA reported a significantly smaller total particle concentration (*P* < 0.0001), indicating presence of contamination in the EV preparation likely by protein aggregates and/or residual EQ solution. No significant differences were found between the mean and mode diameters of unlabeled particles assessed in light scatter mode and labeled EVs assessed by fNTA (*P* > 0.05) [Table 3], but there were differences in the overall PSD, with significant differences in the reported D10 (*P* < 0.001), D50 (*P* = 0.004), D90 (*P* = 0.03), and standard deviation (*P* = 0.005) between conventional NTA and fNTA. 

**Table 3:** NanoSight LM10 analysis of CFS EVs. Summary of EV number and size distribution, with or without the FTLA algorithm, quantified by NanoSight analysis

		**Mean (nm)**	**Mode (nm)**	**SD** **(nm)**	**D10 (nm)**	**D50 (nm)**	**D90** **(nm)**	**Total Concentration (Particles/mL)**	**Number of Completed Tracks**	**Number of Valid Tracks**
Light scatter	FTLA size distribution	196.4 ± 1.7	165.0 ± 3.1	104.2 ± 7.6	106.0 ± 1.2	172.0 ± 1.2	306.0 ± 8.0	7.43E11 ± 1.89E9	26006	8663
	size distribution	194.1 ± 1.1	155.3 ± 2.4	117.7± 5.0	98.0 ± 1.2	168.7 ± 0.7	304.3 ± 2.3	7.43E11 ± 1.89E9	26006	8663
Fluorescent	FTLA size distribution	227.7 ± 5.2	93.7 ± 7.5	228.8 ± 11.3	78.0 ± 2.0	150.7 ± 1.3	469.3 ± 17.4	3.47E11 ± 4.32E9	13839	4704
	size distribution	214.3 ± 6.3	87.0 ± 7.1	235.2 ± 12.2	69.7 ± 1.2	142.7 ± 2.2	404.0 ± 17.8	3.47E11 ± 4.32E9	13839	4704

Mean and SD of three technical measurements for light scatter NTA and fluorescent NTA are shown. Concentrations adjusted for dilution factors. All values provided are batch average results from 3 technical replicates. D10 is the point in the size distribution where 10% of the sample is contained, D50 is the point where 50% of the sample is contained (median), and D90 is the point where 90% of the sample is contained. CFS: Cell-free saliva; EVs: extracellular vesicles; FTLA: finite track length adjustment; SD: standard deviation.

We also performed NTA using a second platform, the ViewSizer 3000, and found results that closely approximated the fNTA [Supplementary Figure 3, Supplementary Table 3]. There were no differences in total particle concentration nor in mean and mode particle diameter comparing the NanoSight LM10 fNTA and ViewSizer 3000 light scatter NTA results (*P* > 0.05). Comparing the overall PSD (D10, D50, D90, standard deviation) between NanoSight fNTA and ViewSizer light scatter NTA, there were significant differences in D50 (*P* = 0.0003) and the standard deviation (*P* = 0.009). Comparing the NanoSight LM10 and ViewSizer 3000 light scatter data, there were no significant differences in mean, mode, D10, D90, or standard deviation in particle size, but the ViewSizer reported significantly larger D50 (*P* = 0.02) and total particle concentration (*P* = 0.012) values. 

### Wes protein analysis of saliva EVs

**Figure 5:**
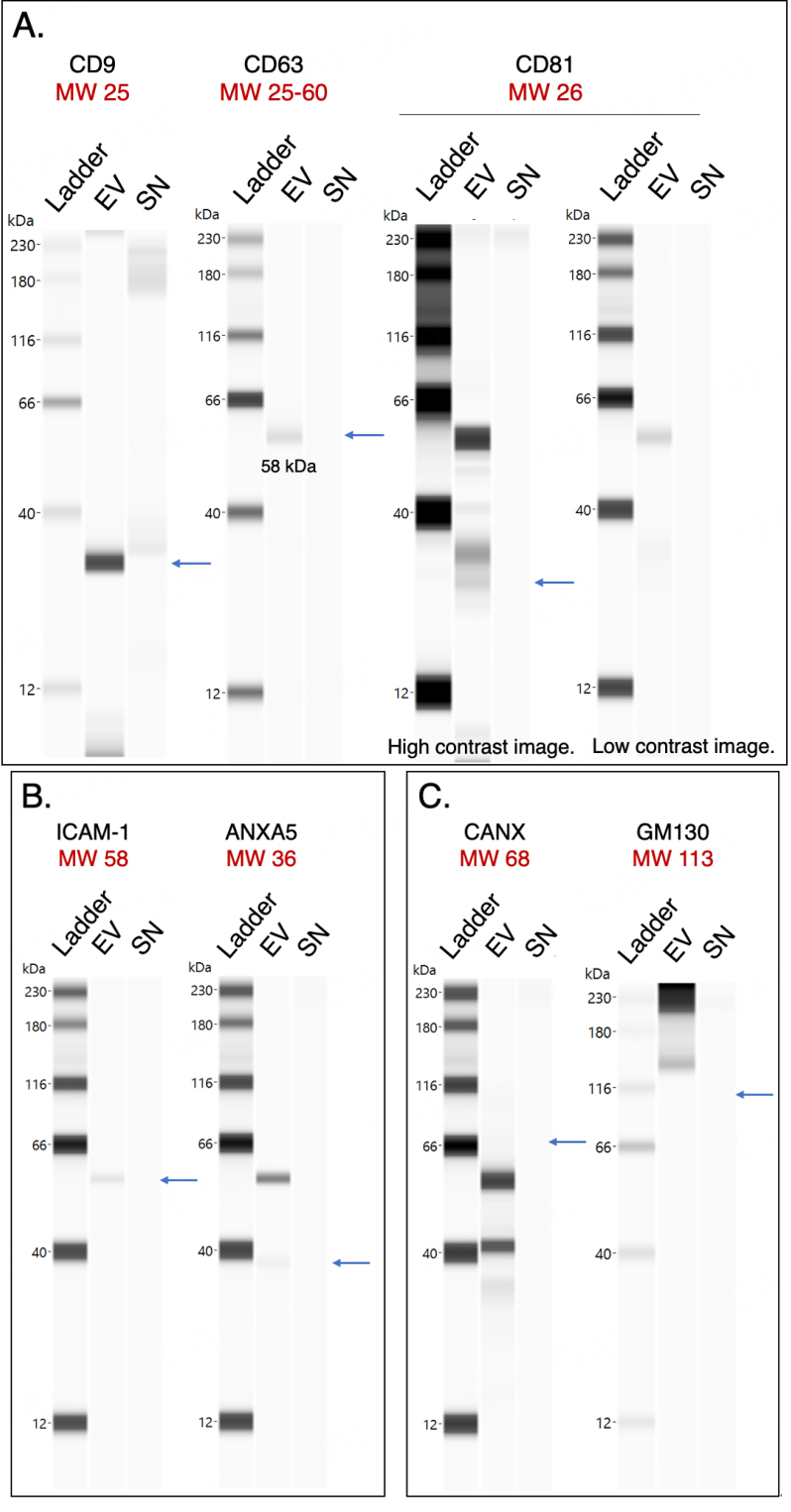
Protein composition of CFS EVs and SN analyzed by the Wes system (ProteinSimple). Calculated molecular weights (in kDa) are shown in red. Pseudo-gel images of representative runs shown for (A) Tetraspanins CD9, CD63, and CD81; (B) Intercellular cell adhesion molecule (ICAM-1) and annexin A5 (ANXA5); and (C) Intracellular proteins calnexin (CANX) and GM130. For all runs, the peak signal-to-noise ratio given by the software ≥ 10. CFS: Cell-free saliva; EVs: extracellular vesicles; MW: molecular weight; EV: pooled extracellular vesicle sample; SN: pooled supernatant sample.

Capillary Western immunoassays performed on the Wes (ProteinSimple, San Jose, CA, USA) were used for protein quantitation^[27]^. The Wes method can detect proteins of various sizes with high sensitivity while only using small amounts of precious biological sample^[28]^. In Wes, proteins are separated by size in capillaries, where they are incubated with primary and horseradish peroxidase (HRP)-conjugated secondary antibodies and a chemiluminescent substrate. The chemiluminescent signal is detected, quantified, and displayed as an electropherogram or virtual blot-like image. Wes analysis verified the presence of the tetraspanin proteins CD9, CD63, and CD81 which were enriched in the EV pellet compared to the SN in varying intensities following isolation with EQ [Figure 5A]. CD81 was enriched in the EV pellet but with low intensity. In addition, the EV-associated proteins ICAM-1 (Intercellular adhesion molecule-1) and ANXA5 (Annexin A5) were detected in the EV pellet but were barely detectable in the SN [Figure 5B]. The lack of calnexin (CANX, endoplasmic reticulum protein) and GM130 (Golgi protein) verified the purity of the EV sample [Figure 5C]. These proteins are major constituents of non-EV structures often co-isolated with EVs. Their absence in the EV prep indicates little cellular contamination^[29]^.

### Surface biomarker characteristics of saliva EVs

#### Biological particle counts

**Figure 6:**
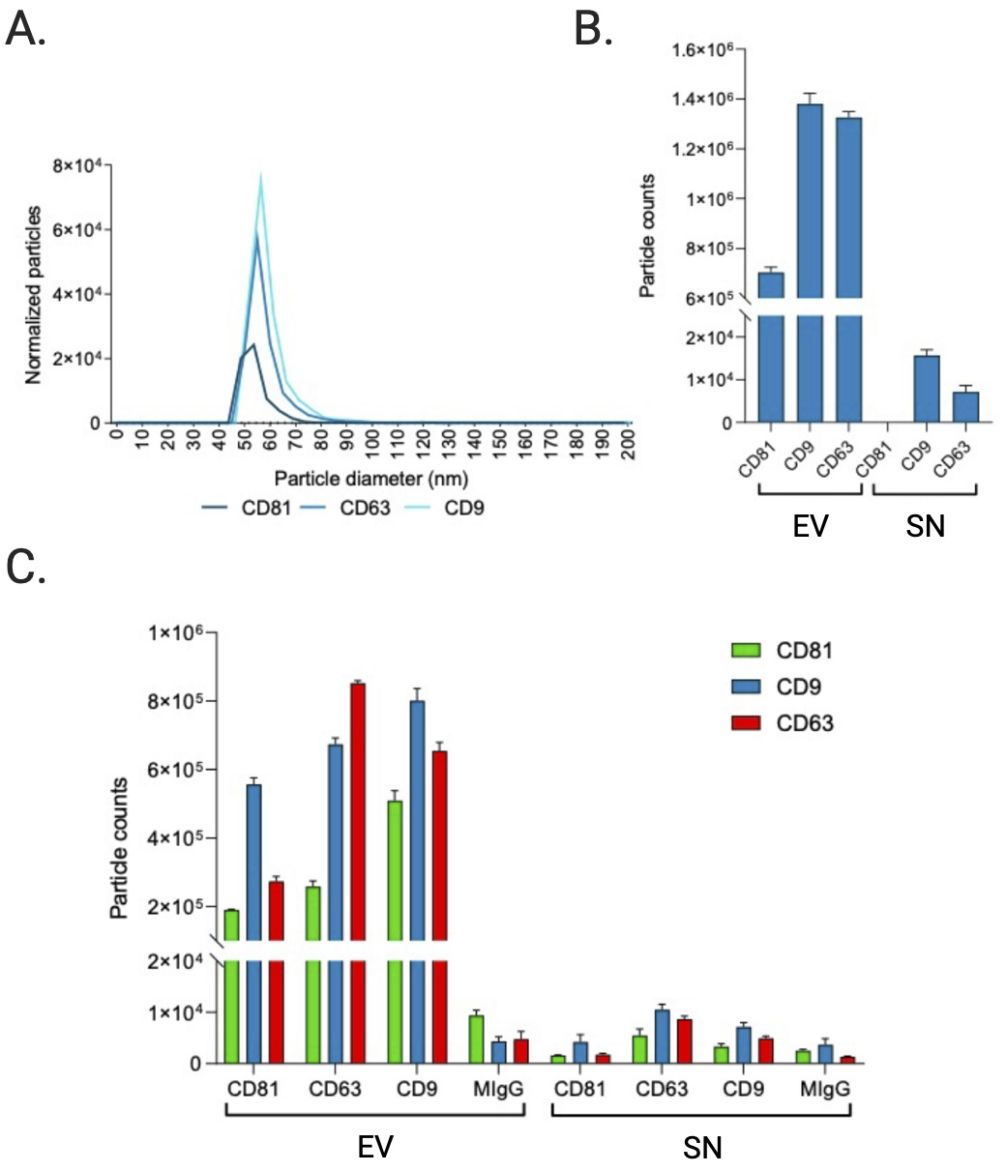
Sizing and enumeration of tetraspanin-positive vesicles. All data has been adjusted for dilution of the sample onto the chip. Average of three technical replicates that were run. Particle number is quantified by the number of particles in a defined area on the antibody capture spot. (A) Interferometry-based sizing and counting of label-free EVs, normalized to MIgG control. Limit of detection is 50-200 nm. (B) Quantification of the number of CD9, CD63, and CD81-positive particles after probing particles with a cocktail of fluorescent tetraspanin antibodies. Any particle that expresses a copy of the target protein and is detected by fluorescence will be counted, regardless of size. (C) Particle count by fluorescent channel shows the number of particles on each capture spot that express each fluorescent marker.

To obtain more elaborate results, the EV and SN samples were analyzed using single particle interferometric reflectance imaging sensing (SP-IRIS) by the ExoView R100 platform (NanoView Biosciences, Boston, MA, USA). Briefly, the ExoView combines microfluidics with immunodetection and interferometric imaging to detect unlabeled EVs based on their expression of EV marker proteins. Here, CD9, CD63, and CD81-positive immuno-captured EVs from the pooled EV sample and pooled SN sample were imaged on a single EV basis and subsequently sized [Table 4] and counted [Figure 6A]. Results show a higher number of tetraspanin-positive vesicles in the EV pellet compared to the SN, as expected since these transmembrane proteins are enriched in EVs. The counts of CD9, CD63, and CD81-positive EVs positively correlated (Pearson’s r = 0.62) with the protein quantitation and relative intensities of the pseudo-gels obtained by Wes capillary immunoassay, including the low signal of CD81 in this EV preparation. However, while the signal intensity of CD9 was significantly greater than that of CD63 in the capillary immunoassay, SP-IRIS measured counts of CD63-positive particles that were nearly equal to the counts of CD9-positive particles. This discrepancy could be due to differences in the CD63 antibody used across assays.

**Table 4:** Sizing of tetraspanin-positive EVs by interferometry-based label-free measurements performed on each capture spot

**Marker**	**Mean (nm)**	**Median (nm)**
CD9	58.2	62.5
	57.9	61.9
CD63	58.2	60.9
	58.1	58.6
CD81	56.0	54.9
	55.9	53.1

Lower limit of detection: 50 nm. Upper limit of detection: 200 nm. Means and medians (each value calculated from three spots for each capture antibody) from two technical replicates are shown. Particle sizing across the two replicates had a deviation of 0.1-0.2 nm.

#### Single particle phenotyping by fluorescence

In addition to direct detection of unlabeled EVs through interferometric analysis, it is also possible to label the bound EVs using secondary fluorescently labeled antibodies and detect EVs with fluorescence. Following sizing and enumeration of unlabeled EVs, the chips were incubated with labeling antibodies consisting of anti-CD9 CF-488, anti-CD63 CF-647, and anti-CD81 CF-555 for 3-color phenotyping of captured particles [Figure 6B-C]. In contrast with SP-IRIS to count unlabeled particles, which has a limit of detection of 50-200 nm diameter, the fluorescent phenotyping counts any particle that expresses a copy of the target protein, regardless of size. Compared to SP-IRIS particle counts, fluorescent phenotyping reported a 17.5-fold increase in the number of CD9-positive vesicles, a 21.6-fold increase in the number of CD63-positive vesicles, and a 35-fold increase in the number of CD81-positive vesicles, indicating there were a large number of particles present outside the 50-200 nm size range for non-fluorescent detection [Figure 6B]. In agreement with the SP-IRIS and immunoassay data, the fluorescent particle counts show that the majority of vesicles expressed CD9 and CD63 [Figure 6C]. Thus, this trend held not only for EVs 50-200 nm in diameter but also for EVs outside of this size range. No signal was detected in the CD81 channel of the SN. Since the majority of vesicles expressed more than one type of tetraspanin, we analyzed this further via colocalization analysis.

**Figure 7:**
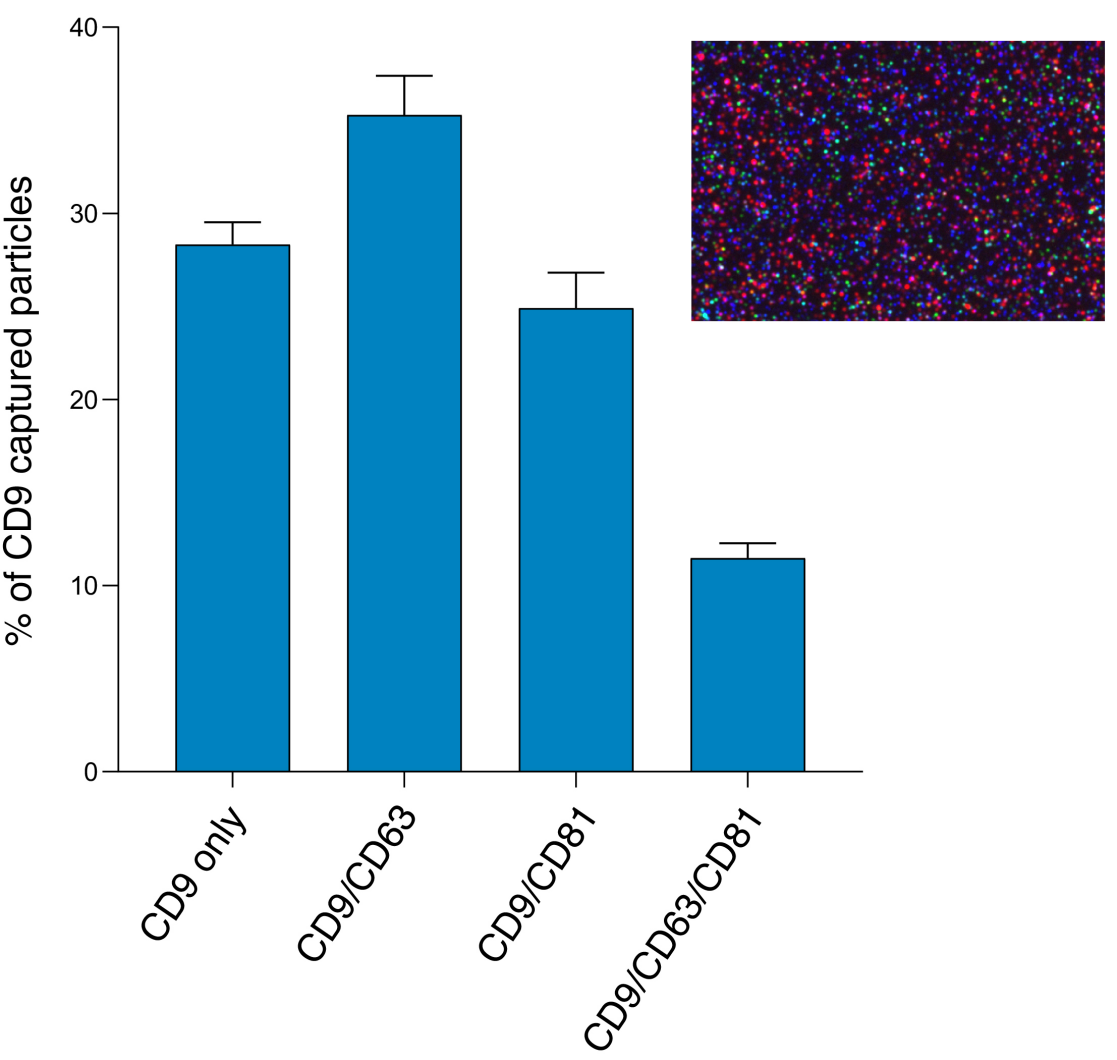
Colocalization of tetraspanins CD9, CD63, and CD81 detected by 3-color fluorescence measured on the ExoView R100. The fluorescent antibodies are CD9 (blue), CD63 (red), and CD81 (green). CD9 capture spot data for the pooled EV sample (average of three technical replicates). Inset shows the CD9 capture spot composite fluorescent image.

We examined co-expression of the three tetraspanins by looking at colocalization of fluorescent signals after analyzing composite images from the different fluorescent channels [Supplementary Figure 4]. The results show that the majority of vesicles (63%) express either CD9 only (28%) or both CD9 and CD63 (35%). A quarter (25%) of vesicles express CD9 and CD81 and 12% express all three tetraspanins [Figure 7].

## DISCUSSION

Saliva is the most accessible and non-invasive biofluid, which makes it a very appealing biofluid to use for the detection of biomarkers of disease, especially among children. Indeed, saliva has been shown to reflect overall health status, containing robust biomarkers (including EV biomarkers) of systemic disease such as cancers and autoimmune diseases^[30,31]^. Saliva also contains biomarkers associated with asthma^[21,32]^. Yet, to our knowledge, saliva EVs from patients with diagnosed asthma have not been investigated so the potential of saliva EVs as biomarkers of asthma, asthma prognoses, and asthma subtypes remains unknown. Here, we demonstrate proof of concept that we can isolate EVs from CFS of patients with asthma by characterizing a pooled EV sample representative of a cohort, SICAS, which is comprised of children with asthma living in an urban area.

We confirmed the presence of EVs using negative-stain TEM. EVs were distinguished from background and non-EV particles due to their distinct morphology and contrast properties. The EVs pooled from 180 individual samples representing the SICAS cohort appeared as electron-dense membranous structures with a mean (median) diameter of 64.95 (55.09) nm. The data were positively skewed by the presence of two microvesicles exceeding 250 nm in size. Thus, EQ isolated different EV subpopulations, with sizes consistent with that of exosomes but also larger vesicles (microvesicles). Note that while TEM is widely used to characterize EVs, the sample preparation for TEM imaging can induce changes in the EV morphology; the “cup-shaped” appearance commonly seen among EVs in TEM micrographs is an artifact of the fixation and contrast steps of the sample prep^[33]^. Thus, cryo-electron microscopy, which shows the lipid bilayer and better preserves EV size, is preferred for visualization of single vesicles^[34-36]^.

Conventional light scatter NTA analysis of EVs measured a total particle concentration of 7.43E11 ± 1.89E9 particles/mL with a mode size of 155.3 ± 2.4 nm (NanoSight LM10), while fNTA results reported a total particle concentration of 3.47E11 ± 4.32E9 particles/mL with a mode size of 87.0 ± 7.1 nm (NanoSight LM10). We interpret the raw NTA and fNTA results rather than FTLA results, as FTLA can introduce artifacts and so the interpretation of these results is not recommended^[37]^. Contrary to TEM which can visualize particles of the smallest sizes, NTA has a larger limit of detection (~50 nm, minimum detectable vesicle sizes ~70-90 nm on Nanosight NS500) due to the small size and relatively low refractive index of EVs, explaining the shifted PSD reported by NTA compared to TEM^[37,38]^. Furthermore, NTA may overestimate EV sizes because it visualizes particles in the native state, compared to TEM which, as mentioned above, can result in dehydration and shrinkage of EVs through the sample preparation process.

Unlike TEM, NTA is a non-specific particle analysis method that cannot differentiate EVs from non-EV structures such as lipoproteins or residual EQ reagent (which was present in our prep as shown in Supplementary Figure 2 and could skew results). NTA of fluorescently labeled EVs attempts to overcome this limitation. Our fNTA analysis of EVs labeled with a fluorescent lipophilic membrane dye reported a significantly smaller total particle concentration compared to conventional NTA and with a mode particle size (87.0 nm) closer to the mode reported by TEM (42.7 nm) than that reported in light scatter mode (155.3 nm). This highlights that vesicle-specific fNTA measurements should be carried out when possible to obtain more robust particle counts and PSDs. However, lacking knowledge of the stain, its optical properties, the method of staining (the fluorescent dye is a proprietary commercially available kit), and a detergent-treated control, we can’t be certain that the fNTA results are specific to intact EVs and as such, our results should be interpreted with caution. Additionally, our particle concentrations cannot be interpreted quantitatively because we lack multiple measurements at different sample dilutions. Regardless, because NTA often overestimates particle concentrations and because the true concentration of an EV sample is unknown, we avoid interpreting the absolute concentration determination and instead shift our focus to the analysis of particle sizes^[25,39]^. 

NTA methods are known to provide accurate results for sizing monodisperse and bimodal reference particles, which we showed here by measuring a 100 nm polystyrene latex sphere standard Supplementary Figure 1, but they fall short regarding accuracy of concentration determination and precision in general^[40]^. Thus, as a general guideline, it is recommended to apply more than one orthogonal method for particle size and concentration measurements of complex polydisperse samples such as EVs. Here, we confirmed our results with measurement on a second NTA instrument, the ViewSizer 3000. While not a truly orthogonal method (e.g., both instruments rely on the Stokes-Einstein equation for particle size determination), the ViewSizer 3000’s optical system makes it unique from other particle tracking instruments such as the NanoSight LM10 because it includes multispectral illumination with three lasers (at 450 nm, 520 nm, and 635 nm) and detection techniques that enable video recording of scattered light from individual particles in multiple spectral bands, allowing measurement of particle sizes even in a highly polydisperse sample. Compared to conventional light scatter on the NanoSight, the ViewSizer reported a lower total particle concentration and larger median particle size, but there were no significant differences in mean and mode particle size nor D10, D90, and standard deviation. Though not significantly different, the right tail of the ViewSizer PSD (D50, D90) was more skewed to higher particle diameters (average ViewSizer D50/D90 across replicates: 198.84/466.95 nm, average NanoSight D50/D90 across replicates: 168.7/304.3 nm). The ViewSizer 3000 measures more closely approximated the fNTA results; the reported particle concentration measures on the ViewSizer were not significantly different from the particle concentrations reported by fNTA. Such differences between the reported EV concentrations and PSDs are caused mainly by differences in the minimum detectable vesicle sizes (i.e., detection limits) across the two instruments, but also on parameters such as instrument settings, device-dependent attributes, and operator bias.

Both NTA instruments measured larger EV diameters compared to TEM, as others have noted^[39]^. This is again due to the fact that TEM sample preparation causes shrinkage, slightly underestimating EV size, but more importantly because smaller EVs are below the limit of detection for NTA. Also, EV membrane surface proteins may impede mobility in solution resulting in inaccurate particle sizing by the Stokes-Einstein equation^[41]^. In our study, the NanoSight results can be considered more reliable than the ViewSizer results due to the greater number of tracked particles and additional technical replicate [Table 3, Supplementary Table 3. Yet, given that the hardware and software of these two NTA platforms differ considerably, as well as the sample movement and standardized settings, the relatively similar particle concentration and PSD results provide higher confidence in our measures of total particle concentration and size, which overall was consistent with the expected size range of EVs.

Capillary Western immunoassay analysis of the EV sample confirmed the presence of the tetraspanins CD9, CD63, and CD81, although CD81 was not greatly expressed. These results were corroborated using SP-IRIS of CD81-positive immuno-captured EVs. This result is likely not due to low CD81 antibody efficiency, as the two methods used different capture antibodies (JS-81 mouse monoclonal antibody for ExoView, rabbit polyclonal antibody with AA 113-201 of recombinant CD81 as the immunogen [accession NP_004347.1] for Wes). This finding is important as many studies use CD81 expression to normalize levels of EV markers of interest^[42,43]^. We also detected the presence of EV-associated proteins ICAM-1 and ANXA5, which were enriched in the EV sample compared to the SN, as expected. ICAM-1, involved in cell signaling events, plays a role in the targeting of EVs to recipient cells, while the lipid-binding protein ANXA5 is involved in membrane transport and fusion. Thus, the proteins described and characterized here are transmembrane proteins and cytosolic proteins with membrane-binding capacity that we expected to be present in the EV sample. The absence of CANX and GM130 (which we did not expect to be enriched in the EVs) in the pooled sample indicated that this EV isolation method did not co-isolate soluble proteins associated with intracellular compartments other than the plasma membrane and endosomes.

In addition to TEM and NTA, we also assessed the EV size and concentration using an emerging technology, SP-IRIS and fluorescent phenotyping on the ExoView R100. The particle counts of antigen-positive EVs in the EV sample and SN sample demonstrate that EQ-purified CFS EVs were significantly enriched in vesicles compared to the EQ SN, as expected, and consisted primarily of CD9 or CD63 positive vesicles. The EV counts in the SN samples were very low and also consisted primarily of CD9 and CD63 positive vesicles. Compared to SP-IRIS for particle counts, which has a dynamic range of 50-200 nm, fluorescent phenotyping reported a much higher number of tetraspanin-positive vesicles. This finding is supported by the TEM and NTA data; TEM detected particles < 50 nm, while NTA (which is unable to detect particles < 50 nm) and TEM both reported the presence of microvesicles > 200 nm. Compared to the fNTA measurements, the ExoView reported significantly smaller particle counts in the EV prep (*P* < 0.001). This may indicate that a sizable portion of EVs in the sample may not express CD9, CD63, or CD81, something that should be taken into consideration when choosing a method for EV normalization in downstream studies. However, the ExoView size measurement is specifically for tetraspanin-positive particles, compared to NTA which will measure any particles present. As there is no perfect purification technique which will remove every non-EV particle, the NTA measurement will always have the potential to be biased by non-EV particles present in the sample. More than likely, the discrepancy in fNTA particle counts and the tetraspanin-positive particle counts measured on the ExoView R100 is mainly because the lipophilic membrane dye is non-specific and non-EV particles are still being counted in fNTA.

The particle sizes of antigen-positive EVs [shown in Figure 6 and Table 4] closely approximate the median and mean EV diameter reported by TEM, although the median is more comparable in this case because the mean TEM particle diameter was skewed by the presence of few large particles exceeding 250 nm, whereas SP-IRIS sizing measurements have a dynamic range of 50-200 nm. This limited dynamic range is because particles smaller than 50 nm in diameter have a contrast which is indistinguishable from the chip background when using the standard 405 nm LED. Particles larger than 200 nm are outside the linear range of the interferometry measurement; when using a 405 nm light source, the relationship between Raman scattering intensity and particle size is nonlinear above 200 nm^[44]^. Our results corroborate previous findings comparing different EV analysis techniques (including NTA, TEM, and SP-IRIS/ExoView), which indicated that NTA consistently overestimates the sizes of particles compared to TEM sizing while in contrast, SP-IRIS/ExoView produced size histograms that closely mirrored the TEM size distribution^[39]^. However, immunocapture and immunofluorescence data rely on the specificity of the antibodies used and brightness/limit of detection of the dyes. While these antibody clones have been extensively validated, readers should be aware of the photophysical properties of dyes and their excitation and emission filters when interpreting such results, which we report in Supplementary Table 2.

In conclusion, EQ can be used to isolate bona fide EVs from CFS. Other studies have also reported the efficiency of EQ in comparison with other isolation methods such as ultracentrifugation^[23,45]^. This proof of concept opens a new avenue for saliva EV research for labs that may not have access to the equipment necessary for other EV isolation methods like ultracentrifugation or flow cytometry^[46]^. While this is the first characterization of saliva EVs from patients with asthma, there are many different methods available to isolate EVs, each of which can greatly impact EV yield and purity, affecting interpretation of downstream analyses. Thus, these results may not be generalizable to studies that isolate CFS EVs using a different method. We suggest performing a thorough characterization of an EV preparation prior to any downstream experiments in order to accurately assess the generalizability of results. Characterization of EVs should be performed by applying multiple complementary analytical methods, because, as demonstrated here, each method has unique limitations and biases.

Saliva EVs from this cohort of children with asthma are 64.95 nm in diameter on average, with a median of 55.09 nm, as reported by TEM, and express mostly CD9 and CD63. We also detected enrichment of ICAM-1 and ANXA5. CD81 was not highly expressed in this sample. Future studies that characterize CFS EVs from individuals should assess whether this pattern persists at the individual sample level and whether it is: a result of the EV isolation method, characteristic of CFS EVs, and/or whether this finding of low CD81 expression is characteristic of CFS EVs from patients with asthma in comparison to healthy controls. Also, CFS EVs from individual patients with asthma can be compared to explore whether EV characteristics correspond with different clinical phenotypes or whether unsupervised clustering of EV proteins and other cargo can be used to identify novel underlying etiologies. In addition to pathologies such as asthma, EV release can further be modified by factors such as cellular stress. Thus, in addition to future research comparing CFS EVs of people with asthma and healthy controls, future studies can explore whether external factors such as environmental tobacco smoke or allergens influence EV characteristics. The CFS in SICAS was collected only at one timepoint, but future investigations with longitudinal saliva collections can investigate whether EV characteristics are stable over time, reflecting an underlying etiology, or whether the EVs are responsive to external factors and, if so, how quickly EVs can change in response to an exposure.

This research, however, is subject to several limitations. First, we only characterized a single total EV sample that was comprised of pooled re-suspended EV pellets from 180 individuals enrolled in SICAS with CFS collected at a single timepoint. SICAS is a cohort of predominantly minority children with asthma living in an urban area [Table 1] and thus the results may not be generalizable to other asthma cohorts. However, minority children living in urban areas experience a greater burden of asthma and greater asthma morbidity so novel biomarkers in this group are needed^[47]^.

Second, we isolated CFS EVs using EQ (System Biosciences, Palo Alto, CA, USA), a polymer-based reagent that isolates EVs after a low-speed centrifugation step. This method results in a greater yield of EVs in comparison with other methods, but with lower purity. EQ samples contain EVs of varying sizes and high portions of salts, polymers, and other contaminants such as lipoproteins^[48]^, although our TEM analysis demonstrated there was very little lipoprotein contamination in the EVs isolated from CFS. There was, however, carryover of the EQ reagent Supplementary Figure 2. However, EV isolation by EQ is simple and fast, only requiring an overnight incubation step and a one-step precipitation that can be completed with a benchtop centrifuge. It can also be used with very little sample volumes, an advantage for studies with limited quantities of precious biospecimens. The ease and use of standard equipment make this approach for EV isolation highly scalable to large cohort studies, although the cost of reagents should be considered.

Third, while we compared the EV pellet to the SN that remained after the EV precipitation using EQ, we were unable to make direct comparisons to untreated CFS to confirm that the EQ-treated CFS is enriched in EVs compared to CFS. Fourth, the pooled EV sample underwent a freeze-thaw cycle prior to NTA, SP-IRIS, and protein quantification after being frozen at -80ºC for 4 months (due to lab closures resulting from the COVID-19 pandemic). While studies have found that the EV concentration and size were relatively stable after a single freeze-thaw cycle and storage up to one year^[49,50]^, more work is needed to understand the possible effect of storage conditions on EVs^[36]^. In this case, storage conditions may have affected the reported EV characteristics.

Fifth, our NTA measurements assessed a single dilution. At least two dilutions should be assessed to increase the effective size range, which is necessary because light scattering from smaller and larger particles differs greatly and thus different settings are needed to detect EVs of different sizes^[37]^. However, our confirmation of NanoSight LM10 NTA data on the ViewSizer 3000, which is equipped with three lasers that can more robustly characterize polydisperse samples, helps to overcome this limitation. Lastly, another limitation is that we calibrated both instruments with polystyrene bead standards, which can result in overestimation of particle concentrations in NTA due to their higher refractive index than EVs. Hollow organosilica beads should ideally be used as a reference for calibration, or alternatively silica beads, as their refractive index is more similar to EVs^[51]^. 

This is the first characterization of CFS EVs from patients with asthma. The strength in our approach is in the characterization of EVs across multiple platforms, including multiple different technologies to identify single EVs (TEM, NTA, SP-IRIS), providing an indication of the heterogeneity of the EV preparation examined. We also provide a general overview of the protein composition of the EV sample using a sensitive and quantitative method optimized for low protein concentrations, assessing several proteins expected to be present in the EV prep (e.g., tetraspanins, transmembrane proteins, and cytosolic proteins with membrane-binding capacity) as well as those not expected to be present (e.g., proteins associated with the endoplasmic reticulum and Golgi)^[27,36]^. Each platform applied here has distinct strengths and limitations. By performing multiple size and concentration measurements and characterizing EVs across different platforms, our results provide an initial assessment of CFS EVs that provide the basis for future studies. 

## DECLARATIONS

### Copyright

© The Author(s) 2021.

## References

[1] (2014). Lancet. Controlling asthma. Lancet.

[2] Zar HJ, Ferkol TW (2014). The global burden of respiratory disease-impact on child health. Pediatr Pulmonol.

[3] Ramratnam SK, Bacharier LB, Guilbert TW (2017). Severe Asthma in Children. J Allergy Clin Immunol Pract.

[4] Dharmage SC, Perret JL, Custovic A (2019). Epidemiology of Asthma in Children and Adults. Front Pediatr.

[5] Wenzel SE (2012). Asthma phenotypes: the evolution from clinical to molecular approaches. Nat Med.

[6] Wu G, Yang G, Zhang R (2015). Altered microRNA Expression Profiles of Extracellular Vesicles in Nasal Mucus From Patients With Allergic Rhinitis. Allergy Asthma Immunol Res.

[7] Levänen B, Bhakta NR, Torregrosa Paredes P (2013). Altered microRNA profiles in bronchoalveolar lavage fluid exosomes in asthmatic patients. J Allergy Clin Immunol.

[8] Mortaz E, Alipoor SD, Varahram M (2018). Exosomes in Severe Asthma: Update in Their Roles and Potential in Therapy. Biomed Res Int.

[9] Sastre B, Cañas JA, Rodrigo-Muñoz JM, Del Pozo V (2017). Novel Modulators of Asthma and Allergy: Exosomes and MicroRNAs. Front Immunol.

[10] Palanisamy V, Sharma S, Deshpande A, Zhou H, Gimzewski J, Wong DT (2010). Nanostructural and transcriptomic analyses of human saliva derived exosomes. PLoS One.

[11] Sharma S, Rasool HI, Palanisamy V (2010). Structural-mechanical characterization of nanoparticle exosomes in human saliva, using correlative AFM, FESEM, and force spectroscopy. ACS Nano.

[12] Lässer C, Alikhani VS, Ekström K (2011). Human saliva, plasma and breast milk exosomes contain RNA: uptake by macrophages. J Transl Med.

[13] Wong DT (2008). Salivary Diagnostics: Amazing as it might seem, doctors can detect and monitor diseases using molecules found in a sample of spit. Am Sci.

[14] Kaczor-Urbanowicz KE, Martin Carreras-Presas C, Aro K, Tu M, Garcia-Godoy F, Wong DT (2017). Saliva diagnostics - Current views and directions. Exp Biol Med (Maywood).

[15] Hyyppä T (1981). Studies on immunologic and inflammatory factors in the saliva and gingiva in patients with asthma. Journal of Clinical Periodontology.

[16] Siegler DI, Citron KM (1974). Serum and parotid salivary IgA in chronic bronchitis and asthma. Thorax.

[17] Hyyppa T (1980). Salivary immunoglobulins in children with asthma. J Periodontal Res.

[18] Brasher GW (1971). Salivary IgA children with atopic diseases. Ann Allergy.

[19] Butz A, Bellin MH, Bollinger ME (2016). Salivary cotinine measurement for all children with persistent asthma: spit matters. Ann Allergy Asthma Immunol.

[20] Negretti F, Casetta P (1990). Remarkable increases of salivary IgE levels in allergic syndromes. Int Arch Allergy Appl Immunol.

[21] Little FF, Delgado DM, Wexler PJ (2014). Salivary inflammatory mediator profiling and correlation to clinical disease markers in asthma. PLoS One.

[22] Phipatanakul W, Koutrakis P, Coull BA (2017). The School Inner-City Asthma Intervention Study: Design, rationale, methods, and lessons learned. Contemp Clin Trials.

[23] Zlotogorski-Hurvitz A, Dayan D, Chaushu G (2015). Human saliva-derived exosomes: comparing methods of isolation. J Histochem Cytochem.

[24] Comfort N, Cai K, Bloomquist TR, Strait MD, Ferrante AW, & Baccarelli AA

[25] Gardiner C, Ferreira YJ, Dragovic RA, Redman CW, Sargent IL (2013). Extracellular vesicle sizing and enumeration by nanoparticle tracking analysis. J Extracell Vesicles.

[26] https://www.r-project.org/index.html.

[27] Harris VM

[28] Chen JQ, Heldman MR, Herrmann MA (2013). Absolute quantitation of endogenous proteins with precision and accuracy using a capillary Western system. Anal Biochem.

[29] Lötvall J, Hill AF, Hochberg F (2014). Minimal experimental requirements for definition of extracellular vesicles and their functions: a position statement from the International Society for Extracellular Vesicles. J Extracell Vesicles.

[30] Aqrawi LA, Galtung HK, Guerreiro EM (2019). Proteomic and histopathological characterisation of sicca subjects and primary Sjögren's syndrome patients reveals promising tear, saliva and extracellular vesicle disease biomarkers. Arthritis Res Ther.

[31] Nonaka T, Wong DT

[32] Wexler PJ, Siqueira WL, Helmerhorst EJ, Blicharz TM, Hayman RB (2009). Saliva Diagnostics in Asthma. Proceedings of the American Thoracic Society.

[33] Colombo M, Raposo G, Théry C (2014). Biogenesis, secretion, and intercellular interactions of exosomes and other extracellular vesicles. Annu Rev Cell Dev Biol.

[34] Szatanek R, Baj-Krzyworzeka M, Zimoch J, Lekka M, Siedlar M, Baran J (2017). The Methods of Choice for Extracellular Vesicles (EVs) Characterization. Int J Mol Sci.

[35] (2018). Niel G, D’Angelo G, & Raposo G. Shedding light on the cell biology of extracellular vesicles. Nat Rev Mol Cell Biol.

[36] Théry C, Witwer KW, Aikawa E (2018). Minimal information for studies of extracellular vesicles 2018 (MISEV2018): a position statement of the International Society for Extracellular Vesicles and update of the MISEV2014 guidelines. J Extracell Vesicles.

[37] van der Pol E, Coumans FA, Grootemaat AE (2014). Particle size distribution of exosomes and microvesicles determined by transmission electron microscopy, flow cytometry, nanoparticle tracking analysis, and resistive pulse sensing. J Thromb Haemost.

[38] (2010). der Pol E, Hoekstra AG, Sturk A, Otto C, van Leeuwen TG, Nieuwland R. Optical and non-optical methods for detection and characterization of microparticles and exosomes. J Thromb Haemost.

[39] Bachurski D, Schuldner M, Nguyen PH (2019). Extracellular vesicle measurements with nanoparticle tracking analysis - An accuracy and repeatability comparison between NanoSight NS300 and ZetaView. J Extracell Vesicles.

[40] Vogel R, Savage J, Muzard J (2021). Measuring particle concentration of multimodal synthetic reference materials and extracellular vesicles with orthogonal techniques: Who is up to the challenge?. J Extracell Vesicles.

[41] Skliar M, Chernyshev VS, Belnap DM (2018). Membrane proteins significantly restrict exosome mobility. Biochem Biophys Res Commun.

[42] Goetzl EJ, Yaffe K, Peltz CB (2020). Traumatic brain injury increases plasma astrocyte-derived exosome levels of neurotoxic complement proteins. FASEB J.

[43] Abner EL, Elahi FM, Jicha GA (2020). Endothelial-derived plasma exosome proteins in Alzheimer's disease angiopathy. FASEB J.

[44] Daaboul GG, Gagni P, Benussi L (2016). Digital Detection of Exosomes by Interferometric Imaging. Sci Rep.

[45] Helwa I, Cai J, Drewry MD (2017). A Comparative Study of Serum Exosome Isolation Using Differential Ultracentrifugation and Three Commercial Reagents. PLoS One.

[46] Erdbrügger U, Lannigan J (2016). Analytical challenges of extracellular vesicle detection: A comparison of different techniques. Cytometry A.

[47] Azmeh R, Greydanus DE, Agana MG (2020). Update in Pediatric Asthma: Selected Issues. Dis Mon.

[48] Gemoll T, Rozanova S, Röder C (2020). Protein Profiling of Serum Extracellular Vesicles Reveals Qualitative and Quantitative Differences After Differential Ultracentrifugation and ExoQuick^TM ^Isolation. J Clin Med.

[49] Jeyaram A, Jay SM (2017). Preservation and Storage Stability of Extracellular Vesicles for Therapeutic Applications. AAPS J.

[50] Yuana Y, Böing AN, Grootemaat AE (2015). Handling and storage of human body fluids for analysis of extracellular vesicles. J Extracell Vesicles.

[51] Varga Z, van der Pol E, Pálmai M Hollow organosilica beads as reference particles for optical detection of extracellular vesicles. J Thromb Haemost.

