# Supplementary material for: Isolation and characterization of extracellular vesicles in saliva of children with asthma
Source: Extracell Vesicles Circ Nucl Acids. 2021 Mar 30;2(1):29–48. doi: 10.20517/evcna.2020.09 (PMC8340923; doi:10.20517/evcna.2020.09)
Supplement: Supplementary file 1 [file evcna-2-1-29-SupplementaryMaterials.pdf]

DOI: 10.20517/2574-209.xxxx.xx

Extracellular Vesicles and Circulating Nucleic Acids

Open Access
Open Access
Check for updates

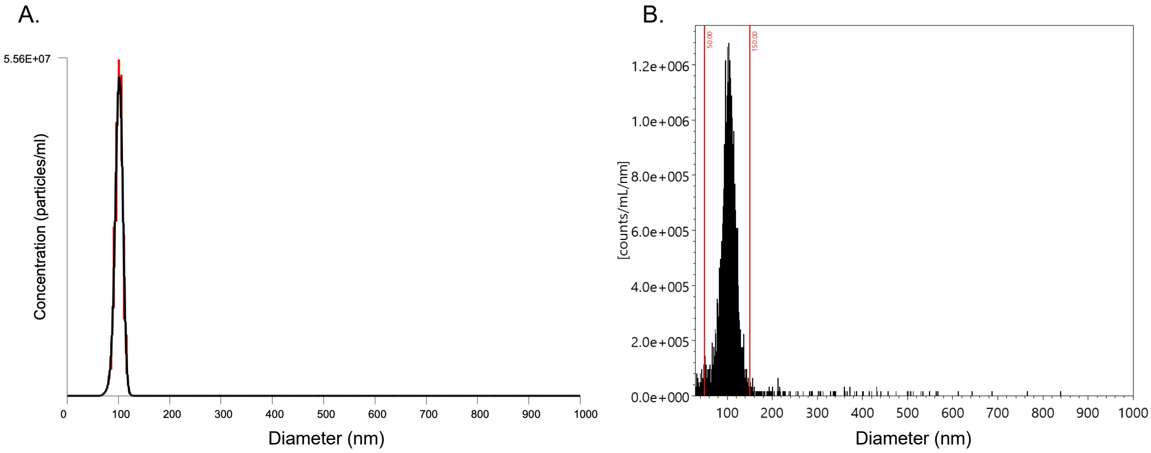

Supplementary Figure 1. Particle size distributions of 100 nm monodisperse polystyrene bead standards on (A) NanoSight LM10 and (B) ViewSizer 3000 verify instrument calibration. (A) NanoSight LM10 FTLA distribution average size/concentration. Mean particle diameter: 100.0 ± 1.4 nm, SD: 22.0 ± 3.1 nm; FTLA mean particle diameter: 101.0 ± 1.0 nm, SD: 22.7 ± 15.3 nm. Red error bars indicate ± standard error of the mean. Results are batch average of three technical replicates. (B) ViewSizer 3000 mean particle diameter: 102 nm, SD: 17 nm. Results averaged from 25 videos (300 frames/video). SD: Standard deviation; FTLA: finite track length adjustment.

© The Author(s) 2021. Open Access This article is licensed under a Creative Commons Attribution 4.0 International License (https://creativecommons.org/licenses/by/4.0/), which permits unrestricted use, sharing, adaptation, distribution and reproduction in any medium or format, for any purpose, even commercially, as long as you give appropriate credit to the original author(s) and the source, provide a link to the Creative Commons license, and indicate if changes were made.

Extracellular Vesicles and Circulating Nucleic Acids

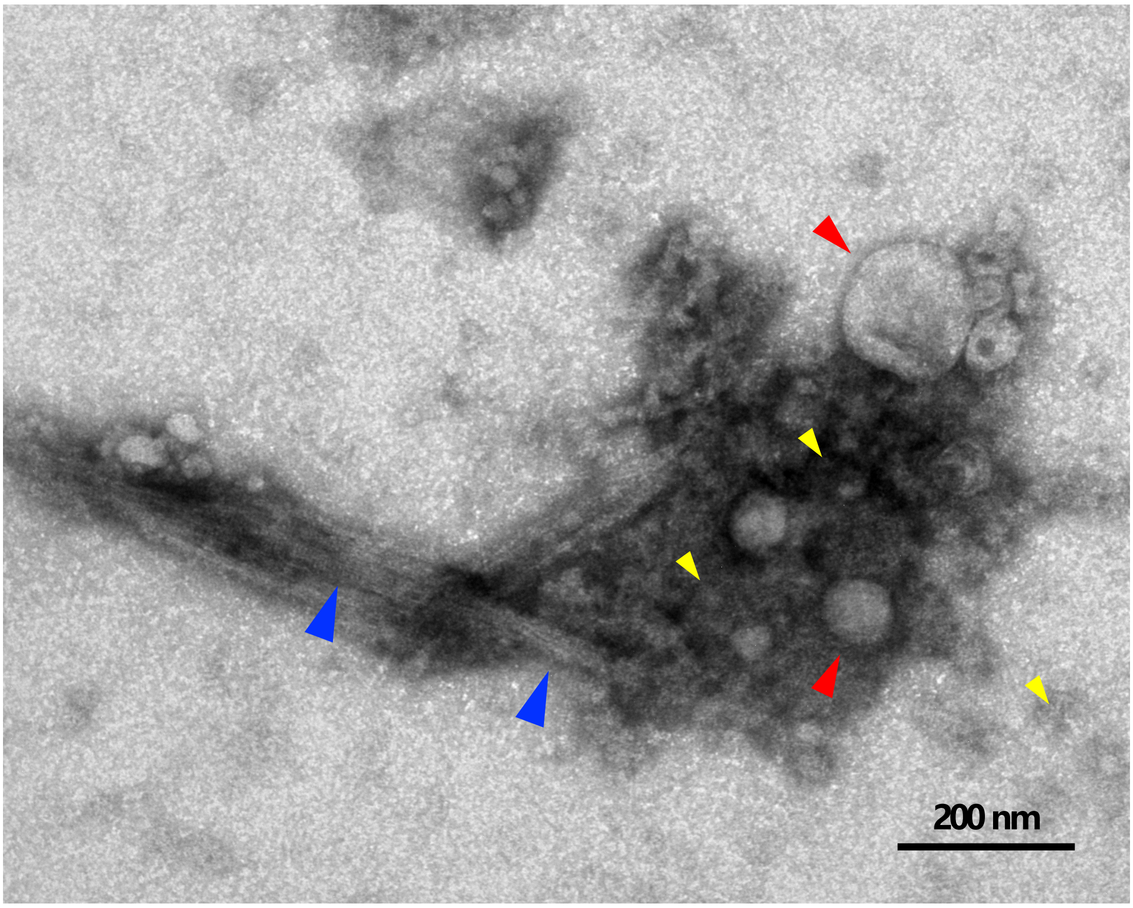

Supplementary Figure 2: Representative electron microscopy negative staining image showing EQ reagent contamination. Electron micrograph shows aggregates of vesicles (red arrowheads). Other unidentified structures seen in the background (yellow arrowheads) could be protein aggregates and/or dense aggregates of EVs. Fibrous-like shapes (blue arrowheads) could result from carry-over of EQ reagent. Scale bar reflects the magnification at the camera. 100k magnification, scale bar 200 nm. EQ: ExoQuick-TC.

© The Author(s) 2021. Open Access This article is licensed under a Creative Commons Attribution 4.0 International License (https://creativecommons.org/licenses/by/4.0/), which permits unrestricted use, sharing, adaptation, distribution and reproduction in any medium or format, for any purpose, even commercially, as long as you give appropriate credit to the original author(s) and the source, provide a link to the Creative Commons license, and indicate if changes were made.

DOI: 10.20517/2574-1209.xxxx.xx
Extracellular Vesicles and Circulating Nucleic Acids

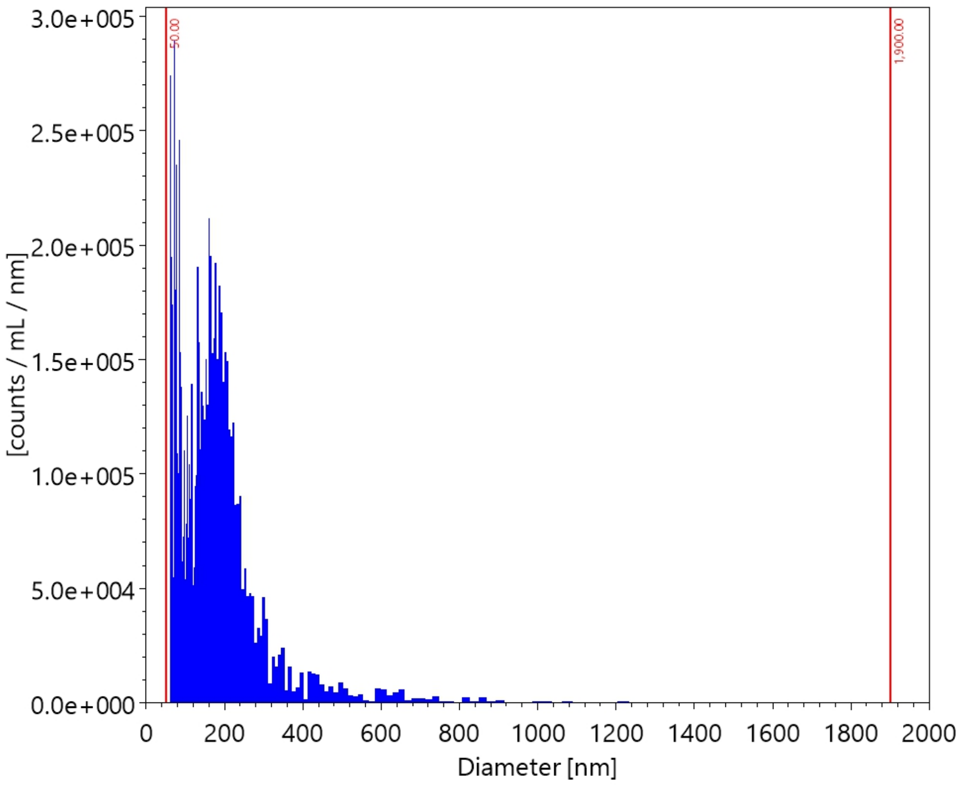

Supplementary Figure 3. Characterization of CFS EVs by NTA using the ViewSizer 3000. Representative PSD of the EV sample measured on an alternate NTA platform, the ViewSizer 3000. Graph is representative for measuring samples using the optimal particle/frame rate according to the ViewSizer 3000. Concentration histogram is not background-subtracted and is unadjusted for the dilution factor. CFS: Cell-free saliva; EVs: extracellular vesicles; PSD: particle size distribution.

© The Author(s) 2021. Open Access This article is licensed under a Creative Commons Attribution 4.0 International License (https://creativecommons.org/licenses/by/4.0/), which permits unrestricted use, sharing, adaptation, distribution and reproduction in any medium or format, for any purpose, even commercially, as long as you give appropriate credit to the original author(s) and the source, provide a link to the Creative Commons license, and indicate if changes were made.

DOI: 10.20517/2574-1209.xxxx.xx
Extracellular Vesicles and Circulating Nucleic Acids

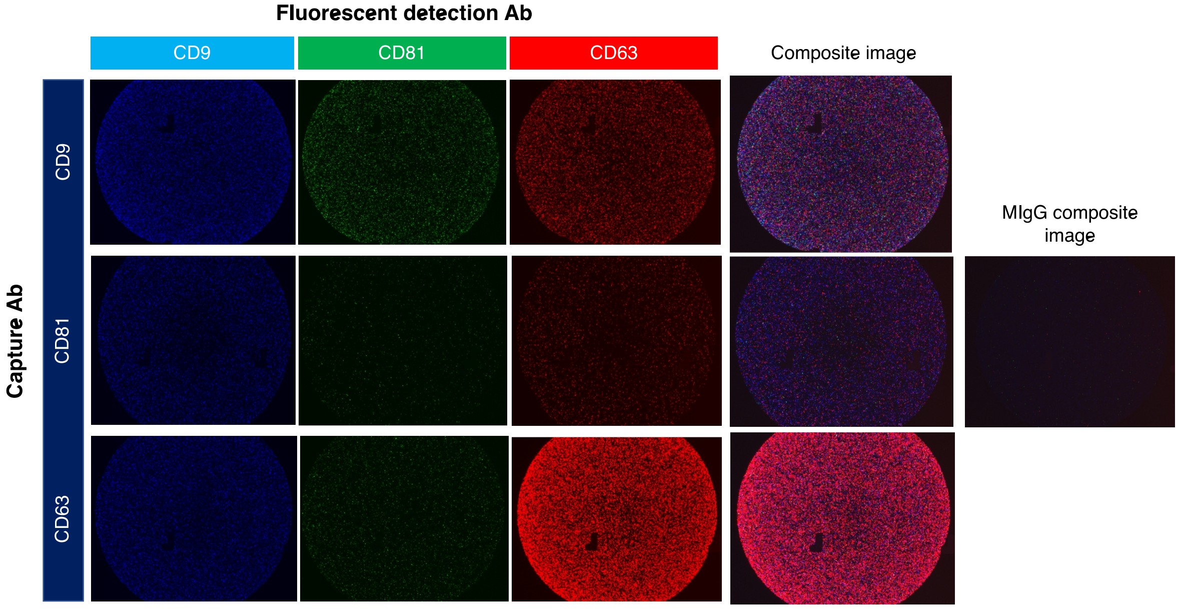

Supplementary Figure 4. Representative image of fluorescent staining in the three fluorescent channels. To further examine immuno-captured EVs with additional phenotyping markers, EVs were labeled with anti-CD9 CF-488, anti-CD81 CF-555, and anti-CD63 CF-647 for 3-color phenotyping. Composite images show colocalization of fluorescent markers for each capture antibody spot. MIgG is the negative control.

© The Author(s) 2021. Open Access This article is licensed under a Creative Commons Attribution 4.0 International License (https://creativecommons.org/licenses/by/4.0/), which permits unrestricted use, sharing, adaptation, distribution and reproduction in any medium or format, for any purpose, even commercially, as long as you give appropriate credit to the original author(s) and the source, provide a link to the Creative Commons license, and indicate if changes were made.

Extracellular Vesicles and Circulating Nucleic Acids

Supplementary Table 1. NanoSight LM10 Capture and Analysis Settings.

Capture Settings		Analysis Settings	
Camera type:	sCMOS	Background Extract:	On
Shutter length:	30 ms* Varied**	Detection Threshold:	4 – Multi
Shutter setting:	1200	Blur:	AUTO
Camera gain:	500	Min track length:	10
Frame rate:	Varied	Min expected size:	AUTO

*Conventional mode; **Fluorescent mode.

© The Author(s) 2021. Open Access This article is licensed under a Creative Commons Attribution 4.0 International License (https://creativecommons.org/licenses/by/4.0/), which permits unrestricted use, sharing, adaptation, distribution and reproduction in any medium or format, for any purpose, even commercially, as long as you give appropriate credit to the original author(s) and the source, provide a link to the Creative Commons license, and indicate if changes were made.

Extracellular Vesicles and Circulating Nucleic Acids

Supplementary Table 2. Immunofluorescence excitation and emission wavelengths.

Channel	LED wavelength (nm)	Excitation wavelength (nm)	Emission wavelength (nm)
‘Red’ (CD63 Ab)	623	625-655	665-725
‘Green’ (CD81 Ab)	567	543-568	580-608
‘Blue’ (CD9 Ab)	470	465-495	505-530

The excitation and emission wavelengths (in nm) of the three fluorescent channels of the ExoView R100 are shown.

© The Author(s) 2021. Open Access This article is licensed under a Creative Commons Attribution 4.0 International License (https://creativecommons.org/licenses/by/4.0/), which permits unrestricted use, sharing, adaptation, distribution and reproduction in any medium or format, for any purpose, even commercially, as long as you give appropriate credit to the original author(s) and the source, provide a link to the Creative Commons license, and indicate if changes were made.

Extracellular Vesicles and Circulating Nucleic Acids

Supplementary Table 3. ViewSizer 3000 analysis of CFS EVs.

Mean (nm)	Mode (nm)	SD (nm)	D10 (nm)	D50 (nm)	D90 (nm)	Total Concentration (Particles/mL)	Total Completed Tracks
194	72	131	94.5	197.2	437.6	2.36E+11	1439
208	169	139	113.2	200.5	496.3	2.12E+11	1342

Summary of EV number and size distribution quantified by the ViewSizer 3000. Individual results of two replicates are shown. Each run is an average of 25 videos (300 frames/video). Total particle concentrations are background-subtracted and adjusted for dilution factors. Data was processed in “LogBinSilica” mode and integrated from 50-1900 nm. D10 is the point in the size distribution where 10% of the sample is contained, D50 is the point where 50% of the sample is contained (median), and D90 is the point where 90% of the sample is contained. SD: Standard deviation; CFS: cell-free saliva; EVs: extracellular vesicles.

© The Author(s) 2021. Open Access This article is licensed under a Creative Commons Attribution 4.0 International License (https://creativecommons.org/licenses/by/4.0/), which permits unrestricted use, sharing, adaptation, distribution and reproduction in any medium or format, for any purpose, even commercially, as long as you give appropriate credit to the original author(s) and the source, provide a link to the Creative Commons license, and indicate if changes were made.
