# Supplementary material for: Isolation and characterization of extracellular vesicles in saliva of children with asthma
Source: Extracell Vesicles Circ Nucl Acids. Author manuscript; Available in PMC 2021 Aug 5. (PMC8340923; doi:10.20517/evcna.2020.09)
Supplement: Supplementary file 1 [file NIHMS1691208-supplement-suppl-figure_1.pdf]

Surnameet al. Extracellular Vesicles and Circulating Nucleic AcidsYear;Volume:Number
DOI: 10.20517/2574-209.xxxx.xx

Extracellular Vesicles and Circulating Nucleic Acids

Open Access
Open Access

Check for updates

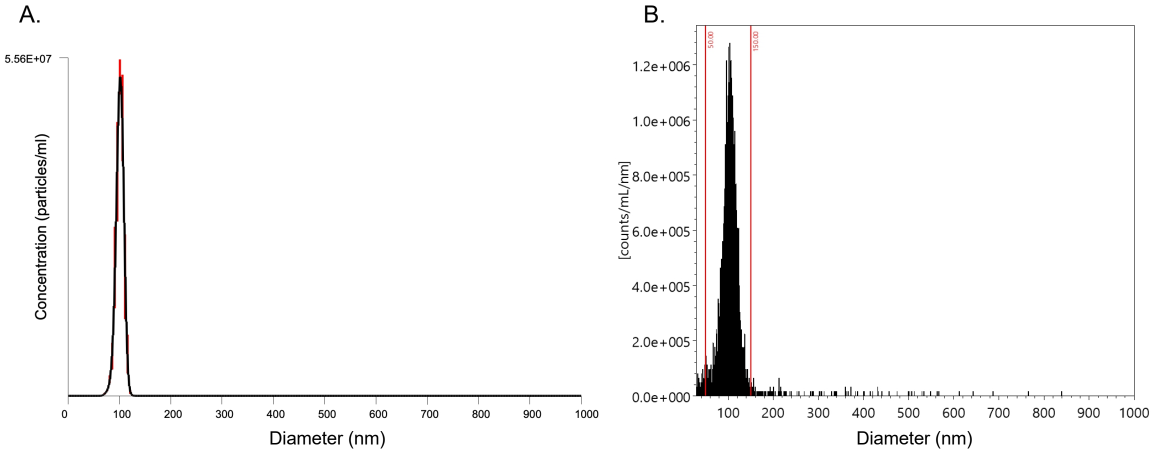

Supplementary Figure 1. Particle size distributions of 100 nm monodisperse polystyrene bead standards on (A) NanoSight LM10 and (B) ViewSizer 3000 verify instrument calibration. (A) NanoSight LM10 FTLA distribution average size/concentration. Mean particle diameter: 100.0 ± 1.4 nm, SD: 22.0 ± 3.1 nm; FTLA mean particle diameter: 101.0 ± 1.0 nm, SD: 22.7 ± 15.3 nm. Red error bars indicate ± standard error of the mean. Results are batch average of three technical replicates. (B) ViewSizer 3000 mean particle diameter: 102 nm, SD: 17 nm. Results averaged from 25 videos (300 frames/video). SD: Standard deviation; FTLA: finite track length adjustment.

© The Author(s) 2021. Open Access This article is licensed under a Creative Commons Attribution 4.0 International License (https://creativecommons.org/licenses/by/4.0/), which permits unrestricted use, sharing, adaptation, distribution and reproduction in any medium or format, for any purpose, even commercially, as long as you give appropriate credit to the original author(s) and the source, provide a link to the Creative Commons license, and indicate if changes were made.
