# Supplementary material for: Isolation and characterization of extracellular vesicles in saliva of children with asthma
Source: Extracell Vesicles Circ Nucl Acids. Author manuscript; Available in PMC 2021 Aug 5. (PMC8340923; doi:10.20517/evcna.2020.09)
Supplement: Supplementary file 2 [file NIHMS1691208-supplement-suppl-figure_2.pdf]

Extracellular Vesicles and Circulating Nucleic Acids

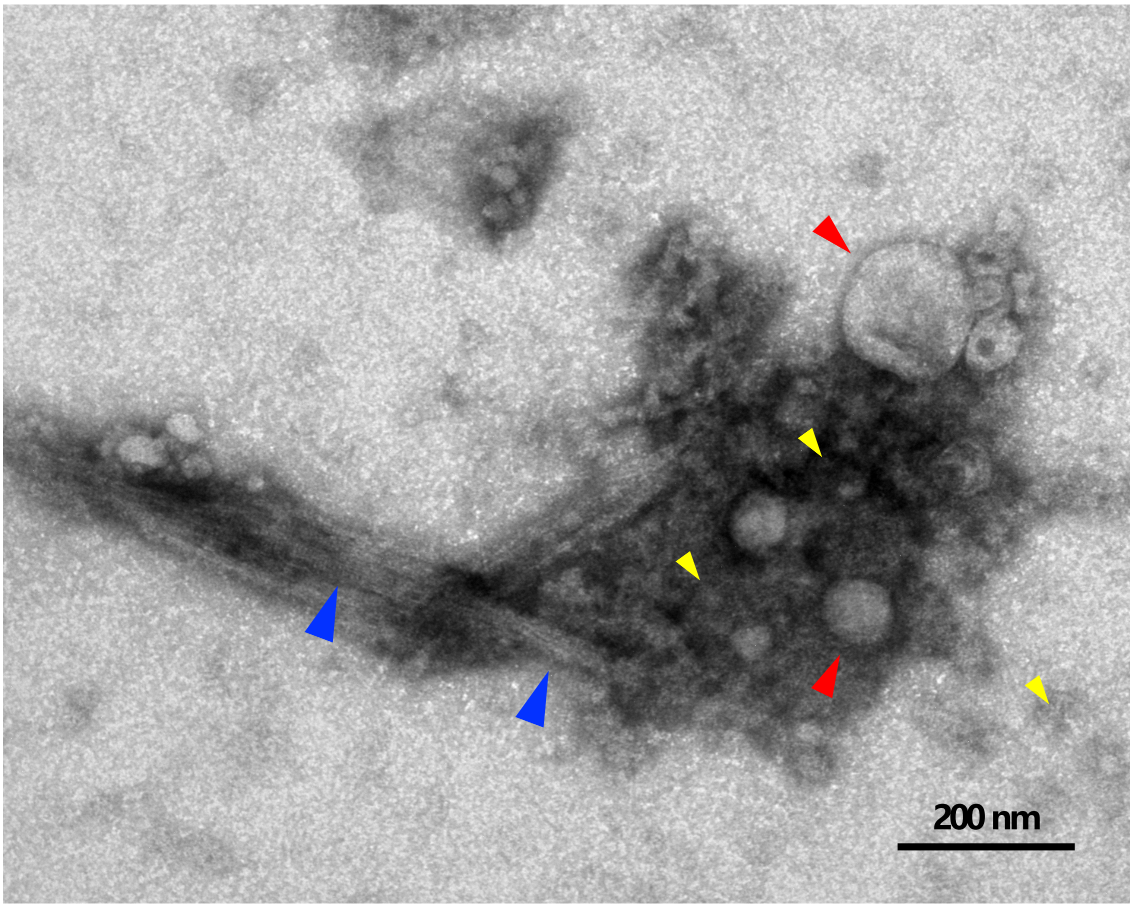

Supplementary Figure 2: Representative electron microscopy negative staining image showing EQ reagent contamination. Electron micrograph shows aggregates of vesicles (red arrowheads). Other unidentified structures seen in the background (yellow arrowheads) could be protein aggregates and/or dense aggregates of EVs. Fibrous-like shapes (blue arrowheads) could result from carry-over of EQ reagent. Scale bar reflects the magnification at the camera. 100k magnification, scale bar 200 nm. EQ: ExoQuick-TC.

© The Author(s) 2021. Open Access This article is licensed under a Creative Commons Attribution 4.0 International License (https://creativecommons.org/licenses/by/4.0/), which permits unrestricted use, sharing, adaptation, distribution and reproduction in any medium or format, for any purpose, even commercially, as long as you give appropriate credit to the original author(s) and the source, provide a link to the Creative Commons license, and indicate if changes were made.
