# Supplementary material for: Isolation and characterization of extracellular vesicles in saliva of children with asthma
Source: Extracell Vesicles Circ Nucl Acids. Author manuscript; Available in PMC 2021 Aug 5. (PMC8340923; doi:10.20517/evcna.2020.09)
Supplement: Supplementary file 3 [file NIHMS1691208-supplement-suppl-figure_3.pdf]

Extracellular Vesicles and Circulating Nucleic Acids

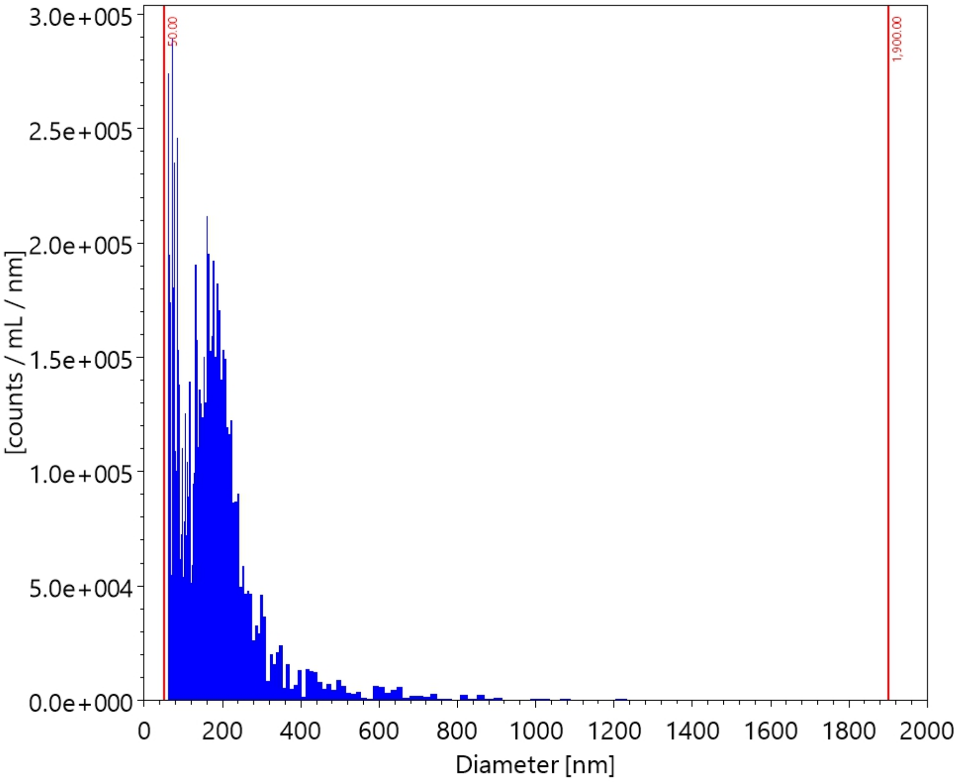

Supplementary Figure 3. Characterization of CFS EVs by NTA using the ViewSizer 3000. Representative PSD of the EV sample measured on an alternate NTA platform, the ViewSizer 3000. Graph is representative for measuring samples using the optimal particle/frame rate according to the ViewSizer 3000. Concentration histogram is not background-subtracted and is unadjusted for the dilution factor. CFS: Cell-free saliva; EVs: extracellular vesicles; PSD: particle size distribution.

© The Author(s) 2021. Open Access This article is licensed under a Creative Commons Attribution 4.0 International License (https://creativecommons.org/licenses/by/4.0/), which permits unrestricted use, sharing, adaptation, distribution and reproduction in any medium or format, for any purpose, even commercially, as long as you give appropriate credit to the original author(s) and the source, provide a link to the Creative Commons license, and indicate if changes were made.
