# Supplementary material for: Isolation and characterization of extracellular vesicles in saliva of children with asthma
Source: Extracell Vesicles Circ Nucl Acids. Author manuscript; Available in PMC 2021 Aug 5. (PMC8340923; doi:10.20517/evcna.2020.09)
Supplement: Supplementary file 4 [file NIHMS1691208-supplement-suppl-figure_4.pdf]

DOI: 10.20517/2574-1209.xxxx.xx

Extracellular Vesicles and Circulating Nucleic Acids

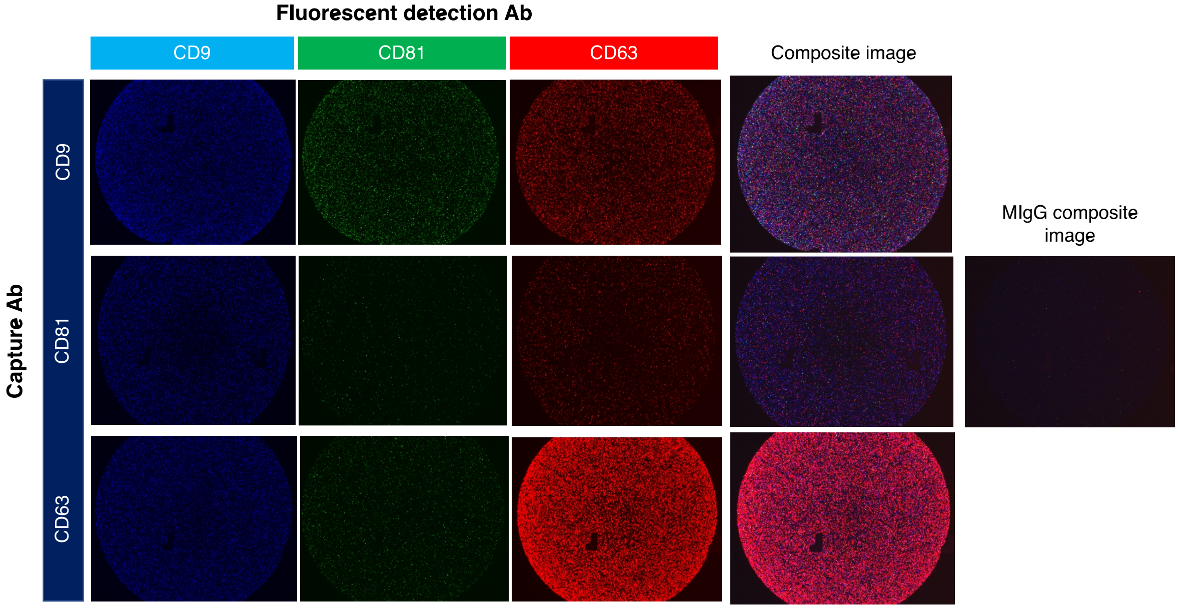

Supplementary Figure 4. Representative image of fluorescent staining in the three fluorescent channels. To further examine immuno-captured EVs with additional phenotyping markers, EVs were labeled with anti-CD9 CF-488, anti-CD81 CF-555, and anti-CD63 CF-647 for 3-color phenotyping. Composite images show colocalization of fluorescent markers for each capture antibody spot. MIgG is the negative control.

© The Author(s) 2021. Open Access This article is licensed under a Creative Commons Attribution 4.0 International License (https://creativecommons.org/licenses/by/4.0/), which permits unrestricted use, sharing, adaptation, distribution and reproduction in any medium or format, for any purpose, even commercially, as long as you give appropriate credit to the original author(s) and the source, provide a link to the Creative Commons license, and indicate if changes were made.
