# Supplementary material for: Isolation and characterization of extracellular vesicles in saliva of children with asthma
Source: Extracell Vesicles Circ Nucl Acids. Author manuscript; Available in PMC 2021 Aug 5. (PMC8340923; doi:10.20517/evcna.2020.09)
Supplement: Supplementary file 5 [file NIHMS1691208-supplement-suppl-table_1.pdf]

Extracellular Vesicles and Circulating Nucleic Acids

Supplementary Table 1. NanoSight LM10 Capture and Analysis Settings.

Capture Settings		Analysis Settings	
Camera type:	sCMOS	Background Extract:	On
Shutter length:	30 ms* Varied**	Detection Threshold:	4 – Multi
Shutter setting:	1200	Blur:	AUTO
Camera gain:	500	Min track length:	10
Frame rate:	Varied	Min expected size:	AUTO

*Conventional mode; **Fluorescent mode.

© The Author(s) 2021. Open Access This article is licensed under a Creative Commons Attribution 4.0 International License (https://creativecommons.org/licenses/by/4.0/), which permits unrestricted use, sharing, adaptation, distribution and reproduction in any medium or format, for any purpose, even commercially, as long as you give appropriate credit to the original author(s) and the source, provide a link to the Creative Commons license, and indicate if changes were made.
