# Supplementary material for: Isolation and characterization of extracellular vesicles in saliva of children with asthma
Source: Extracell Vesicles Circ Nucl Acids. Author manuscript; Available in PMC 2021 Aug 5. (PMC8340923; doi:10.20517/evcna.2020.09)
Supplement: Supplementary file 6 [file NIHMS1691208-supplement-suppl-table_2.pdf]

Extracellular Vesicles and Circulating Nucleic Acids

Supplementary Table 2. Immunofluorescence excitation and emission wavelengths.

Channel	LED wavelength (nm)	Excitation wavelength (nm)	Emission wavelength (nm)
‘Red’ (CD63 Ab)	623	625-655	665-725
‘Green’ (CD81 Ab)	567	543-568	580-608
‘Blue’ (CD9 Ab)	470	465-495	505-530

The excitation and emission wavelengths (in nm) of the three fluorescent channels of the ExoView R100 are shown.

© The Author(s) 2021. Open Access This article is licensed under a Creative Commons Attribution 4.0 International License (https://creativecommons.org/licenses/by/4.0/), which permits unrestricted use, sharing, adaptation, distribution and reproduction in any medium or format, for any purpose, even commercially, as long as you give appropriate credit to the original author(s) and the source, provide a link to the Creative Commons license, and indicate if changes were made.
