# Supplementary material for: Isolation and characterization of extracellular vesicles in saliva of children with asthma
Source: Extracell Vesicles Circ Nucl Acids. Author manuscript; Available in PMC 2021 Aug 5. (PMC8340923; doi:10.20517/evcna.2020.09)
Supplement: Supplementary file 7 [file NIHMS1691208-supplement-suppl-table_3.pdf]

DOI: 10.20517/2574-1209.xxxx.xx
Extracellular Vesicles and Circulating Nucleic Acids

Supplementary Table 3. ViewSizer 3000 analysis of CFS EVs.

Mean (nm)	Mode (nm)	SD (nm)	D10 (nm)	D50 (nm)	D90 (nm)	Total Concentration (Particles/mL)	Total Completed Tracks
194	72	131	94.5	197.2	437.6	2.36E+11	1439
208	169	139	113.2	200.5	496.3	2.12E+11	1342

Summary of EV number and size distribution quantified by the ViewSizer 3000. Individual results of two replicates are shown. Each run is an average of 25 videos (300 frames/video). Total particle concentrations are background-subtracted and adjusted for dilution factors. Data was processed in “LogBinSilica” mode and integrated from 50-1900 nm. D10 is the point in the size distribution where 10% of the sample is contained, D50 is the point where 50% of the sample is contained (median), and D90 is the point where 90% of the sample is contained. SD: Standard deviation; CFS: cell-free saliva; EVs: extracellular vesicles.

© The Author(s) 2021. Open Access This article is licensed under a Creative Commons Attribution 4.0 International License (https://creativecommons.org/licenses/by/4.0/), which permits unrestricted use, sharing, adaptation, distribution and reproduction in any medium or format, for any purpose, even commercially, as long as you give appropriate credit to the original author(s) and the source, provide a link to the Creative Commons license, and indicate if changes were made.
